# NF-κB inhibition prevents acute shear stress-induced inflammation in the saphenous vein graft endothelium

**DOI:** 10.1038/s41598-020-71781-6

**Published:** 2020-09-15

**Authors:** Alexander O. Ward, Gianni D. Angelini, Massimo Caputo, Paul C. Evans, Jason L. Johnson, M. Saadeh Suleiman, Robert M. Tulloh, Sarah J. George, Mustafa Zakkar

**Affiliations:** 1 Bristol Medical School, University of Bristol, Research Floor Level 7, Queens’ Building, Bristol Royal Infirmary, Upper Maudlin Street, Bristol, BS2 8HW UK; 2 Department of Infection, Immunity and Cardiovascular Diseases, University of Sheffield, Sheffield, S10 2TN UK

**Keywords:** Cell adhesion, Cell signalling, Cellular imaging, Mechanisms of disease, Microscopy, Cardiovascular biology

## Abstract

The long saphenous vein (LSV) is commonly used as a conduit in coronary artery bypass grafting. However, long term patency remains limited by the development of vascular inflammation, intimal hyperplasia and accelerated atherosclerosis. The impact of acute exposure of venous endothelial cells (ECs) to acute arterial wall shear stress (WSS) in the arterial circulation, and the subsequent activation of inflammatory pathways, remain poorly defined. Here, we tested the hypothesis that acute exposure of venous ECs to high shear stress is associated with inflammatory responses that are regulated by NF-κB both in-vitro and ex-vivo. Analysis of the LSV endothelium revealed that activation of NF-κB occurred within 30 min after exposure to arterial rates of shear stress. Activation of NF-κB was associated with increased levels of CCL2 production and enhanced binding of monocytes in LSVECs exposed to 6 h acute arterial WSS. Consistent with this, ex vivo exposure of LSVs to acute arterial WSS promoted monocyte interactions with the vessel lumen. Inhibition of the NF-κB pathway prevented acute arterial WSS-induced CCL2 production and reduced monocyte adhesion, both in vitro and in human LSV ex vivo*,* demonstrating that this pathway is necessary for the induction of the acute arterial WSS-induced pro-inflammatory response. We have identified NF-κB as a critical regulator of acute endothelial inflammation in saphenous vein in response to acute arterial WSS. Localised endothelial-specific inhibition of the NF-κB pathway may be beneficial to prevent vein graft inflammation and consequent failure.

## Introduction

Ischaemic heart disease (IHD) is one of the leading causes of mortality in the UK and worldwide^1^. Coronary artery bypass grafting (CABG) remains the gold standard intervention in the presence of complex coronary disease, diabetes and poor ventricular function^2–6^. The long saphenous vein (LSV) is the most used conduit in many patients^7,8^, however, its use is complicated by considerable rates of late stenosis or occlusion due to the development of intimal hyperplasia (IH) and superimposed atherosclerosis^9^. IH is a chronic inflammatory process that starts with endothelial cell (EC) activation, followed by the migration and proliferation of smooth muscle cells (SMC) to the intima, which is then accompanied by alterations in extracellular matrix^10^ (ECM). Vascular inflammation is regulated by signalling intermediaries, including Nuclear Factor-κB, NF-κB (p65), that trigger EC expression of chemokines such as monocyte chemotactic protein-1 (CCL2) and other pro-inflammatory molecules.

The susceptibility of vessels to inflammatory processes may, in part, be related to the vascular bed from which they originate and the types of haemodynamic environment to which they are chronically exposed^11^. Venous ECs in situ, are adapted to chronic levels of low shear stress and, following grafting, they become exposed to shear stresses (defined as force/wall area and measured in dyn/cm^2^) up to tenfold higher in the arterial system^12^. Acute onset of increased rates of shear stress is known to alter the phenotype of venous EC; however, the mechanisms that underpin this response have not been fully elucidated. To gain better insight into the intrinsic molecular mechanisms underlying pro-inflammatory responses of veins to grafting, we investigated the impact of acute shear changes on NF-κB activation and associated vECs response in vitro, ex vivo and in vivo.

## Results

### Acute arterial WSS induces pro-inflammatory responses in LSV ECs ex vivo

**Figure 1:**
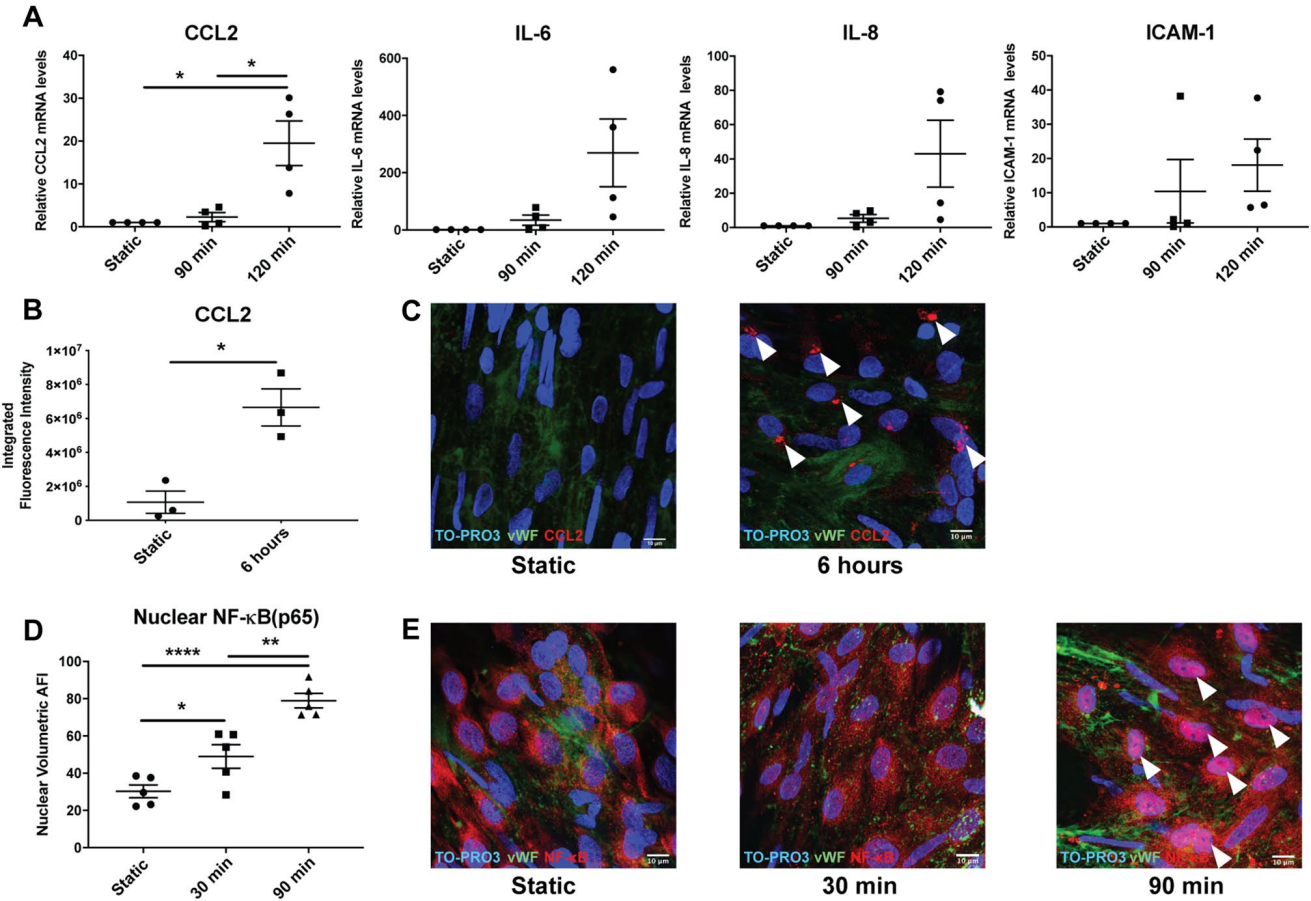
Activation of the LSV endothelium by acute arterial wall shear stress. The LSV endothelium was activated following exposure to acute arterial rates of shear stress, shown by NF-κB translocation and increased CCL2 protein levels. LSV sections were exposed to acute arterial WSS at 12 dyn/cm^2^ for 30 and 90 min or maintained in static conditions and immunostained *en face* for the localisation of NF-κB in LSV ECs. (**A**) LSV portions were exposed to acute arterial WSS for 90 and 120 min or maintained in static conditions, following shear exposure EC RNA was extracted and mRNA levels of pro-inflammatory genes (CCL2, IL-6, IL-8 and ICAM-1) were evaluated by RT-qPCR. Data shown represents independent experiments with 4 different segments of LSV. (**B**) Mean arbitrary fluorescence intensity of CCL2 immunostaining calculated using FIJI. (**C**) Representative images of independent experiments with 3 different segments of LSV, white arrows indicate perinuclear localisation of CCL2 and scale bar represents 10 µm. (**D**) Mean arbitrary fluorescence intensity of NF-κB *en face* immunostaining in endothelial nuclei 3D volumes shown for all 3 conditions, as calculated by Imaris. (**E**) Representative images of independent experiments with 5 different segments of LSV immunostained *en face* for the quantification of nuclear NF-κB, scale bar represents 10 µm. Two-tailed two-sample t-test (**B**) and One-way ANOVA followed by post-hoc pairwise comparisons with Bonferroni correction (**D**) were used to calculate significance, where, **** indicates p < 0.0001, ** indicates p < 0.01 and * indicates p < 0.05.

Freshly harvested segments of human LSV were acutely exposed to arterial shear stress, ex vivo, at 12 dyn/cm^2^, a rate which has previously been associated with EC activation and vascular remodelling in ex vivo venous bypass graft models^12,13^. We noted a significant increase in CCL2 mRNA levels in LSV ECs following exposure to acute high shear stress (acute arterial WSS) (Fig. 1A). E*n face* immunofluorescent staining revealed that exposure of veins to acute arterial WSS increased CCL2 expression in ECs at the protein level (Fig. 1B,C and Supplementary videos 2 and 3).

### NF-κB inhibition prevented a pro-inflammatory response to acute arterial WSS in LSV endothelium

**Figure 2:**
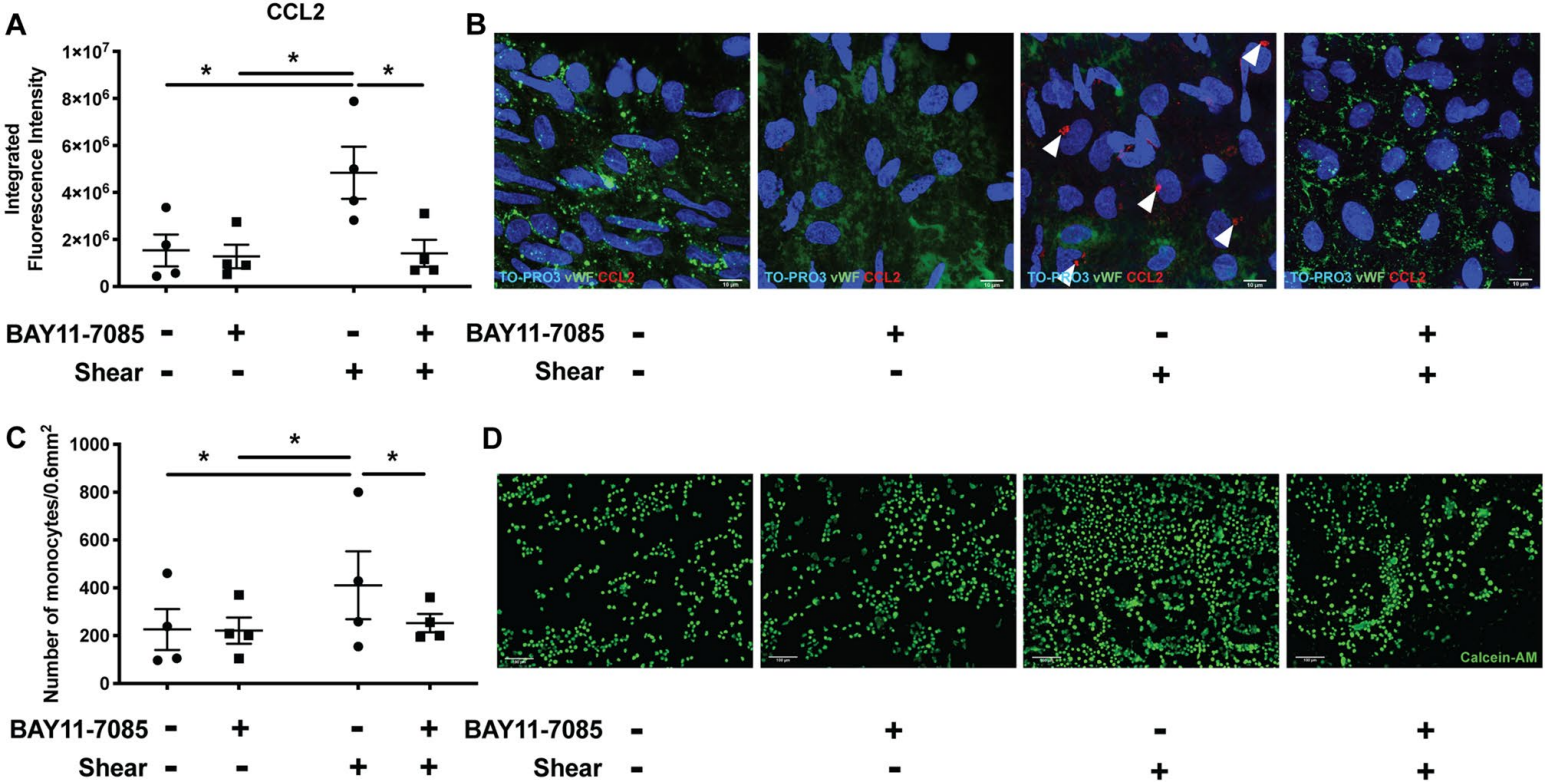
Inhibition of NF-κB and endothelial pro-inflammatory response to acute high shear stress—ex vivo*.* Inhibition of NF-κB prevented the pro-inflammatory response associated with acute arterial WSS in LSV ECs ex vivo. (**A**) Segments of LSV were incubated for 3 h with 50 µmol/L NF-κB inhibitor, BAY11-7085, or 0.5% (v/v) DMSO control and were then exposed to acute arterial WSS for 6 h, or maintained in static conditions. Segments were then immunostained *en face* for the quantification of CCL2 in LSV ECs. (**A**) Mean arbitrary fluorescence intensity of CCL2 immunostaining calculated using FIJI. (**B**) Representative images of independent experiments with 4 different segments of LSV, white arrows indicate perinuclear localisation of CCL2 and scale bar represents 10 µm. Sections of LSV were exposed pre-treated for 3 h with 50 µM NF-κB inhibitor, BAY11-7085, or 0.5% (v/v) DMSO control and were then exposed to acute arterial WSS for 6 h. Following acute arterial WSS exposure, LSV sections were immediately prepared *en face* and incubated with 1 × 10^6^ Calcein-labelled monocytes per mL for 15 min then imaged immediately. (**C**) Total number of monocytes was calculated using CellProfiler software. (**D**) Images represent independent experiments with 4 different segments of LSV and scale bar represents 50 µm. Two-way ANOVA followed by post-hoc pairwise comparisons with Bonferroni correction (**A**,**C**) was used to calculate significance, where, * indicates p < 0.05.

To investigate the possible role of NF-κB, we analysed the expression of p65 NF-kB sub-units in LSVs exposed to arterial shear stress. acute arterial WSS enhanced p65 nuclear translocation in ECs (Fig. 1D,E and Supplementary video 1), indicating that acute arterial shear stress activates NF-kB in LSV endothelium. To investigate the function of NF-κB on pro-inflammatory responses to acute arterial WSS, a selective small-molecule NF-kB inhibitor, BAY11-7085 (BAY) was used (Supplementary Figure 9). The pre-treatment of LSV with 50 µmol/L of BAY modulated EC responses to acute arterial WSS by reducing the levels of CCL2 (Fig. 2A,B). Furthermore, NF-κB inhibition reduced acute arterial WSS induced monocyte adhesion to the LSV endothelium (Fig. 2C,D). Thus, we conclude that NF-κB inhibition can suppress the initiation of monocyte adhesion to ECs in veins under acute arterial WSS.

### Acute arterial WSS activates pro-inflammatory responses and the NF-κB classical pathway in vitro

**Figure 3:**
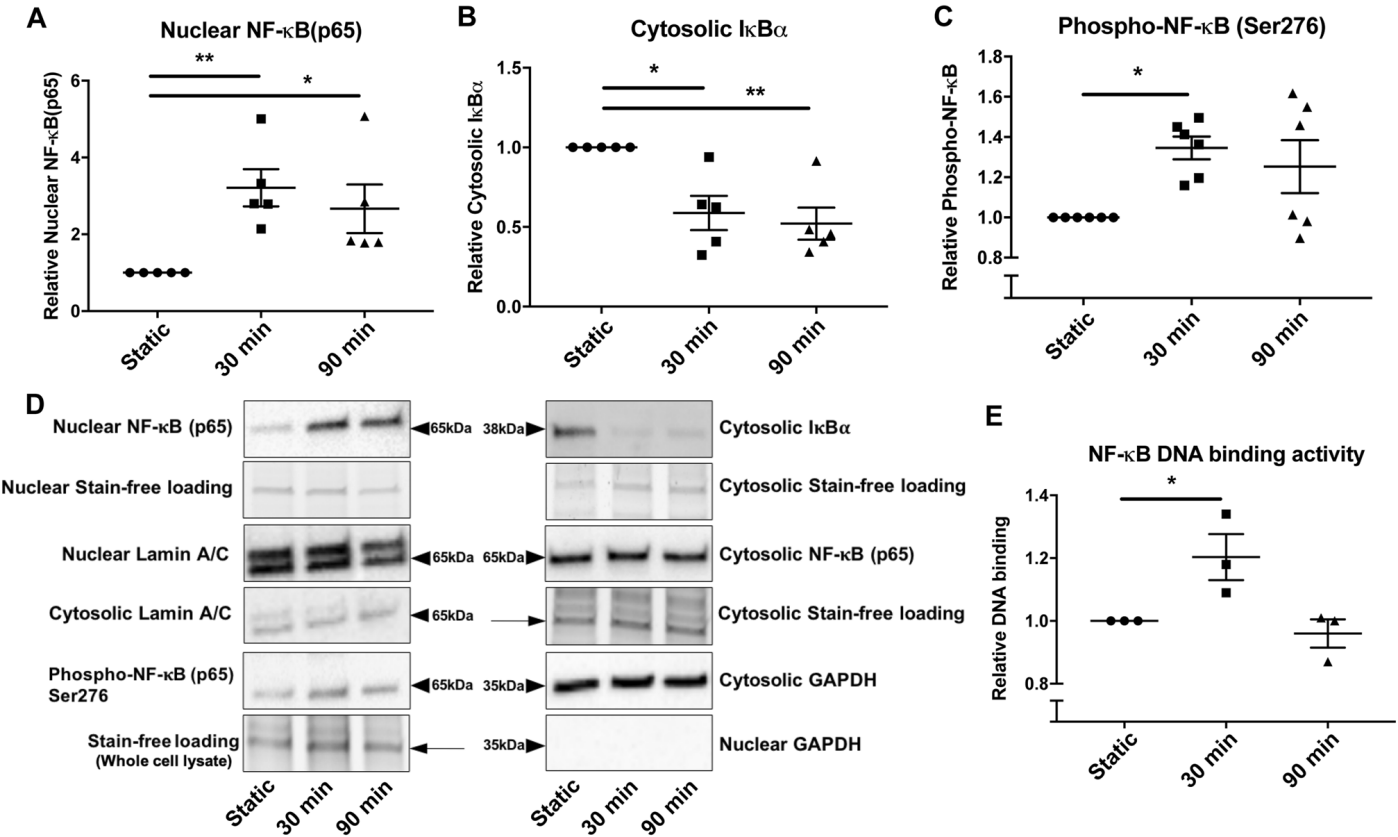
Acute high shear stress and activation of the NF-κB classical pathway—in vitro*.* The NF-κB classical pathway was activated under acute high shear stress as indicated firstly by the nuclear translocation of NF-κB and reciprocal reduction in cytosolic levels of IκB⍺, as well as phosphorylation and increased DNA binding affinity. HUVECs were exposed to acute arterial WSS for 30 and 90 min or maintained in static conditions, then cellular fractions were collected to be analysed by Western blotting. Total amounts of protein were quantified relative to stain-free loading controls and expressed as a fold change of static control. (**A**) Nuclear NF-κB, (**B**) cytosolic levels of IκB⍺ and (**C**) Phospho-NF-κB (Serine residue 276 (Ser276)) from whole cell lysates were analysed by WB, and normalised to stain-free loading controls from nuclear, cytosolic and whole cell lysates, respectively. Western blots shown are representative of 5 or 6 independent experiments showing, in addition to NF-κB and IκB⍺, markers of nuclear and cytosolic fractions, Lamin A/C and GAPDH respectively (**D**). The transcriptional activation of NF-κB under acute arterial WSS, for both 30 and 90 min, was assessed by phosphorylation at Serine residue 276 (**C**) and DNA binding activity (**E**) in whole cell lysates. ELISA based DNA binding activity assay was performed using total cell lysates and was expressed relative to static controls and were representative of 3 independent experiments. One-way ANOVA followed by post-hoc pairwise comparisons with Bonferroni correction was used to calculate significance, where, ** indicates p < 0.01 and * indicates p < 0.05.

**Figure 4:**
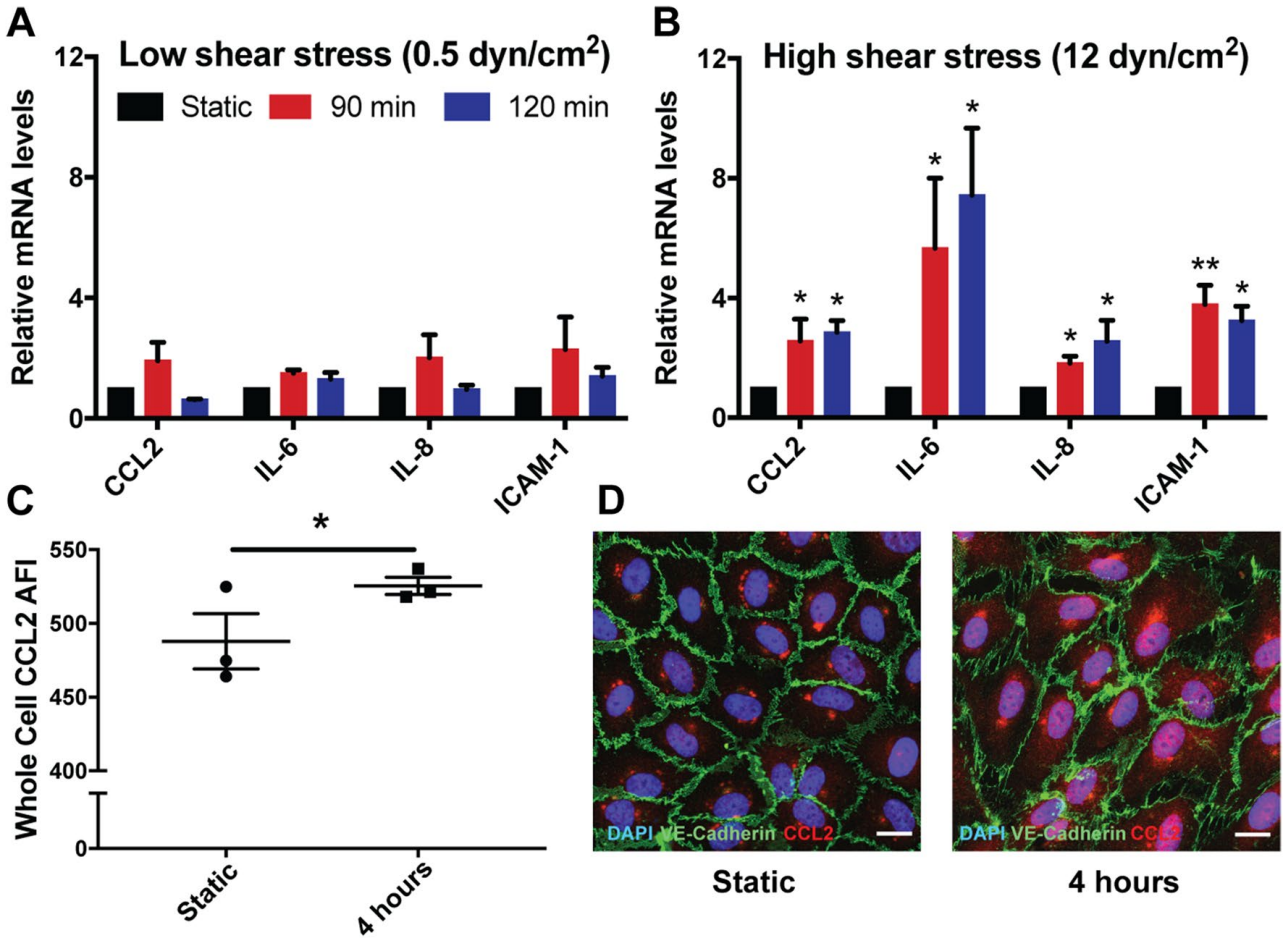
NF-κB target gene response to acute high shear stress—in vitro. Pro-inflammatory, NF-κB target genes were increased in ECs exposed to acute arterial WSS, in vitro. (**C**,**D**) HUVECs were exposed to AASS at 12 dyn/cm^2^ (**A**) and acute low shear stress (ALSS) at 0.5 dyn/cm^2^ (**B**) for 90 and 120 min in order to compare pro-inflammatory gene transcript levels by RT-qPCR. Data represent 6 independent experiments. (**C**,**D**) HUVECs were exposed to AASS at 12 dyn/cm^2^ for 4 h or maintained in static conditions, then immunostained for quantification of total CCL2 using CellProfiler. (**C**) Mean arbitrary fluorescence intensity of CCL2 staining was evaluated in whole cells. (**D**) Images are representative of 3 independent experiments and scale bar represents 15 µm. One-way ANOVA followed by post-hoc pairwise comparisons with Bonferroni correction (**A**,**B**) and two-tailed two-sample t-test (**C**) were used to calculate significance, where, * indicates p < 0.05.

**Figure 5:**
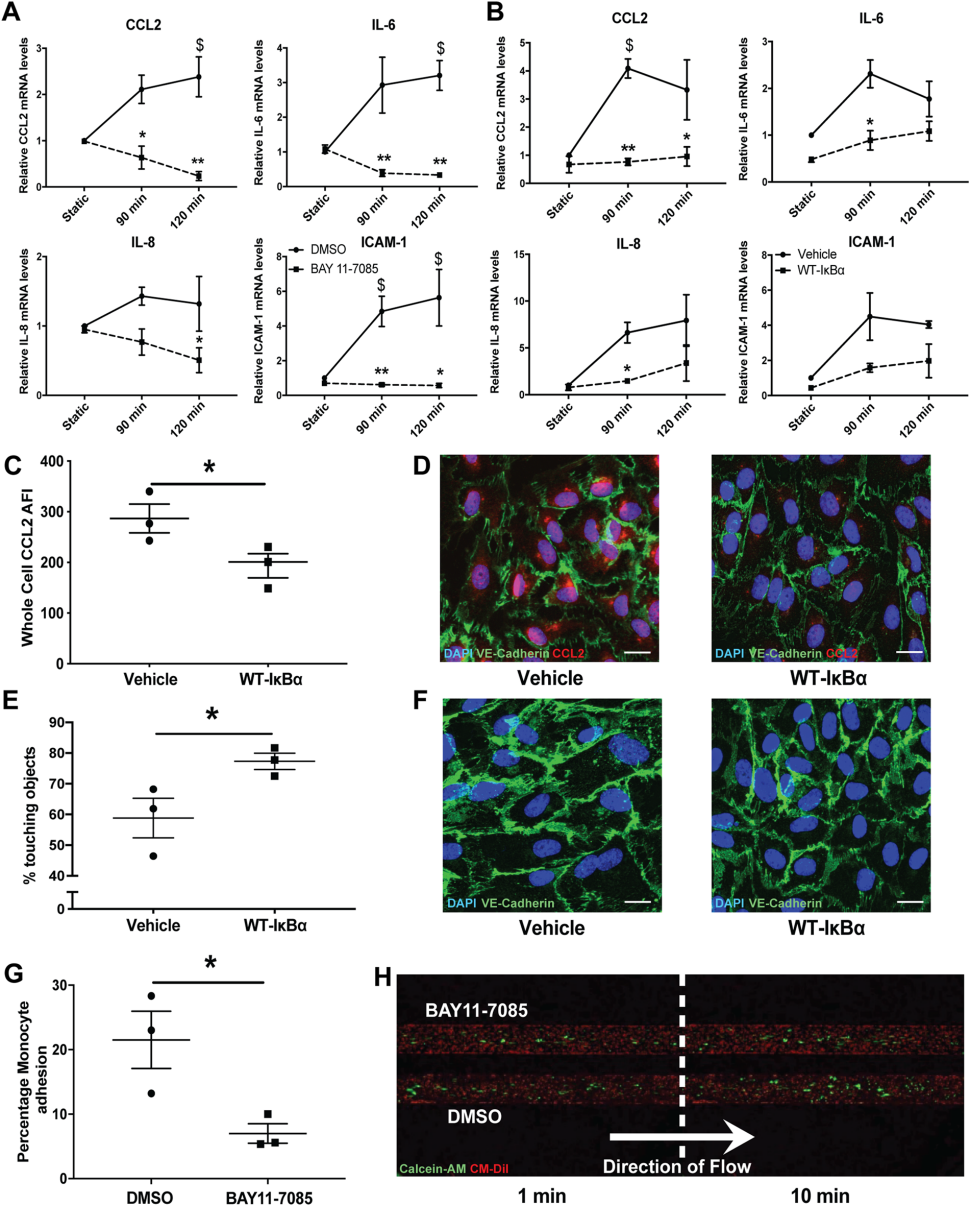
Inhibition of NF-κB and endothelial pro-inflammatory response to acute arterial WSS—in vitro. Inhibition of NF-κB prevented the pro-inflammatory response associated with acute arterial WSS in ECs in vitro*,* as shown by pro-inflammatory mRNA expression, CCL2 protein levels, EC-monocyte interactions and VE-Cadherin cell–cell contact. HUVECs were pre-treated for 1 h with 20 µmol/L NF-κB inhibitor, BAY11-7085 (represented as squares and dashed lines), or 0.2% (v/v) DMSO control (represented as circles and solid lines) and were then exposed to acute arterial WSS for 90 and 120 min or maintained in static conditions. (**A**) mRNA levels of pro-inflammatory genes (CCL2, IL-6, IL-8 and ICAM-1) were then analysed by RT-qPCR. HUVECs were infected with Adenoviruses expressing WT-IκB⍺ transgene (represented as squares and dashed lines) or empty vector viral control (rAd66; represented as circles and solid lines) at 1 × 10^8^ pfu/mL for 18 h, then cultured for a further 48 h and finally exposed to acute arterial WSS for 90 and 120 min (**B**) or 4 h (**C**) and compared to static controls. Adenoviral-mediated overexpression of WT-IκB⍺ was firstly evaluated at the transcript level by RT-qPCR (**B**) and, secondly, at the protein level, by immunocytochemical analysis of CCL2 mean arbitrary fluorescence intensity (**C**,**D**). All data and images represent 3 independent experiments and scale bar represents 15 µm. (**E**,**F**) HUVECs were infected with Adenoviruses expressing WT-IκB⍺ transgene or empty vector viral control (rAd66) at 1 × 10^8^ pfu/mL for 18 h, then cultured for a further 48 h and finally exposed to acute arterial WSS for 4 h or maintained in static conditions then analysed with immunocytochemistry for VE-Cadherin. (**E**) VE-Cadherin-based EC cell–cell contact was measured as a percentage of objects in contact with one another, representing a surrogate marker of barrier disruption. (**F**) Images are representative of 3 independent experiments and scale bar represents 15 µm. (**G**,**H**) HUVECs were pre-treated for 3 h with 20 µmol/L NF-κB inhibitor, BAY11-7085, or 0.2% (v/v) DMSO control and were then exposed to pulsatile acute arterial WSS at 12 dyn/cm^2^ and 1 Hz for 6 h using the integrated, microfluidic capillary-based Bioflux 200 system. Following acute arterial WSS exposure, 3 × 10^6^ Calcein-labelled monocytes per mL, were flowed over the endothelial monolayer and imaged in real time (temporal schematic represented in (**F**), scale bar represents 100 µm). (**G**) Number of monocytes adhered to ECs were enumerated after 10 min and represented as a percentage of the total number of ECs in the channel, data represent 3 independent experiments. Two-way ANOVA followed by post-hoc pairwise comparisons with Bonferroni correction (**A**,**B**) and two-tailed two-sample t-test (**C**,**E**,**G**) were used to calculate significance, where, * indicates p < 0.05, ** indicates p < 0.01 vs. untreated control samples (equivalent time-points), ^$^ indicates p < 0.05 vs. untreated static control.

To validate our ex vivo findings and further investigate the molecular basis involved in the venous EC response to arterial rates of shear stress, we studied the effects of acute arterial WSS on HUVECs in vitro. Acute high shear stress significantly increased nuclear translocation of p65 (Fig. 3A,D) which was associated with reciprocally decreased IκBα expression (Fig. 3B,D) indicating the activation of the NF-κB classical pathway. Furthermore, NF-κB was phosphorylated at Serine residue 276 (Ser-276) following 30 min of acute arterial WSS (Fig. 3C,D), whilst also showing increased DNA binding to the NF-κB consensus oligonucleotide (Fig. 3E), both of which are indicative of transcriptional activation of NF-κB. This transcriptional activation was transient, with levels peaking at 30 min, which is likely indicative of the feed-forward mechanism of negative regulation of NF-κB by IκBα following 60 min acute arterial WSS exposure^13^. We observed that acute arterial WSS, but not low shear, was associated with up regulation of CCL2 (Fig. 4A–D). The suppression of the NF-κB classical pathway using 20 μmol/L BAY pre-treatment resulted in reduced pro-inflammatory activation, similar to what was observed ex vivo (Fig. 5A).

We further validated these findings using adenoviral-mediated over-expression of WT-IκBα (Supplementary Figure 9), which demonstrated that the overexpression of WT-IκBα prevented induction of CCL2 following exposure to acute arterial WSS (Fig. 5B). Moreover, immunoanalysis showed a significant reduction in the expression of CCL2 following 4 h of acute arterial WSS, as compared with static controls (Fig. 5C,D). Whilst, overexpression of WT-IκBα prevented the loss of VE-Cadherin cell–cell contacts following acute arterial WSS exposure (Fig. 5E,F), indicative of an intact endothelial barrier.

We next looked at the impact of NF-κB inhibition on monocyte-EC interactions in vitro, dynamically and in real-time, following exposure to acute arterial WSS*.* We noted that the pre-treatment of HUVECs with 20 μmol/L BAY significantly reduced acute arterial WSS-induced dynamic monocyte adhesion to the vEC monolayer (Fig. 5G,H).

### NF-κB pathway-related gene expression was promoted by acute arterial WSS in vivo

Having observed a significant increase in CCL2 mRNA and protein in the LSV endothelium, we then analysed a publicly available gene expression microarray dataset, generated from a rabbit bilateral interposition vein graft model^14^. Grafts were maintained under low or high shear stress, for 2 and 24 h each. Log expression values (Log Fold Change (LogFC)) of differentially expressed genes (1602 genes) significantly different (p < 0.01) from baseline, in any of the 4 graft conditions were hierarchically clustered and plotted (Supplementary Figure 8A). Interestingly, gene set enrichment analysis between the two acute groups (high and low shear) revealed highly significant enrichment of biological process GO-terms involved in the inflammatory response, cytokine-mediated signalling and neutrophil activity, in grafts exposed to high shear, but not low (Supplementary Figure 8B,C). In addition, the high shear-exposed grafts showed the least overlap with any other graft condition, in terms of both gene overlap and GO-terms, suggesting that grafts exposed to high shear had the largest functionally unique change of any other condition (Supplementary Figure 8D,E). Within grafts exposed to high shear stress for 2 h, significantly up-regulated genes with a LogFC > 1.5 (a total of 114 genes) were analysed further for functional pathway and motif enrichment. Transcriptional regulatory inference, performed using LISA^15^, ranked NF-κB as the most probable transcriptional regulator of this gene-set, which was further supported by the observation that the NF-κB motif was highly enriched within the promoter regions of these genes (Supplementary Figure 8F,G). Finally, KEGG and REACTOME pathway analysis revealed that this gene list was strongly associated with the TNFα- and NF-kappaB signalling pathways, further supporting the role for NF-κB activation in vein grafts following acute shear stress exposure (Supplementary Figure 8H).

## Discussion

Vein graft disease following CABG remains a problem of serious clinical significance despite decades of surgical advances^16,17^. Haemodynamics influence the inflammatory process by controlling leukocyte margination and adhesive interactions and by generating shear stress, which in turn alters EC physiology^18,19^. Increased shear stress rates in the LSV graft have long been considered to directly affect graft patency and survival; however, at present, there is no clinical consensus as to the extent of this effect, perhaps due to the diverse range of rates of graft WSS exposure, postoperatively^20,21^. Furthermore, the molecular cascades that occur after acute increases in WSS rates in LSV graft ECs remain poorly understood. We, and others, have previously shown that arterial and venous EC respond differently to acute high shear stress, in both in vivo and ex vivo graft models^13,22–24^. Here, we show that the NF-κB classical pathway is activated in the endothelium of LSV in response to acute exposure to arterial rates of shear stress and is a critical regulator of vascular inflammation in veins. In vitro experiments further demonstrated that targeting NF-κB prior to acute flow induction was sufficient to prevent endothelial CCL2 expression and monocyte recruitment, as well as EC cell–cell contact disruption, another hallmark of the dysfunctional endothelium.

These findings in the LSV were further substantiated by evidence from analysis of a previously published^14^ in vivo rabbit vein graft model and DNA microarray. Differential expression analysis revealed significantly up-regulated NF-κB and inflammatory pathway-related genes between acute high and low shear-exposed vein grafts. Together, these data provide a possible mechanism for the regulation of acute vein graft inflammation, a factor which is inextricably linked to downstream pathologies. Furthermore, owing to the unique window of opportunity to locally pre-treat the autologous vein graft prior to implantation, inhibition of NF-κB may represent an exciting possibility in the resolution of vascular inflammation within the graft.

Acute onset of increased rates of shear stress is known to promote inflammatory transcriptional responses in ECs in vitro, a process mediated through NF-κB, p38-MAPK and activating protein 1 (AP-1)^17,24–27^. However, no studies so far have directly addressed the role of acute shear stress on NF-κB pathway activation in the vein graft endothelium.

Our findings with regards to activation of CCL2 are in concordance with previously published small animal in vivo data^28,29^. Several previous studies have shown that in vivo, local NF-κB inhibition, using either a decoy cis-element (κB-oligodeoxynucleotide), siRNA-mediated knockdown or an alternate gene therapy approach to overexpress IκBα, limits IH development in multiple vein graft models^30–32^. However, all such previous models propose the same conclusions for their observed results; that it is due to the actions of NF-κB inhibition on reduction of medial VSMC proliferation and migration, and not the role of NF-κB in the acute adaptive response of the vein graft to its new haemodynamic environment. Following this acute haemodynamic adaptation, ECs typically promote endogenous mediators that promote quiescence and dampen inflammation, through the activation of KLF2 and Nrf2 and repression of NF-κB, within 12 h of high WSS exposure^33^. Our novel findings demonstrate a potential role of acute NF-κB inhibition in maintaining this quiescent, anti-inflammatory endothelium prior to and under conditions of arteriovenous transposition that may further help to ameliorate the pathogenic progression to late-stage graft failure.

In conclusion, we identified that acute arterial WSS is responsible for early pro-inflammatory activation of the LVG EC, a process regulated by NF-κB (p65) activation, resulting in upregulation of pro-inflammatory mediators and increased monocyte recruitment, thus, providing the first evidence for the mechanistic involvement of NF-κB in shear-induced inflammation in the vein graft endothelium.

## Methods

Detailed descriptions of all materials and methods can be found in the Supplementary Methods.

### Ex vivo perfusion of veins

The study was approved by the NRES Committee East of England—Norfolk ethics number (REC14/EE/1097) and all use of human tissue conformed to the principles outlined in the Declaration of Helsinki. Surplus segments of surgically prepared human long saphenous vein (LSV), resected during coronary artery bypass graft surgery, from anonymised consenting patients, were exposed to acute arterial WSS, at 12 ± 0.2 dyn/cm^2^, for between 30 min to 6 h using an in-house designed bioreactor system.

### *En face* immunostaining

Briefly, segments of LSV were assessed for the cellular localisation of NF-κB and quantity of CCL2 after exposure to acute arterial WSS by immunostaining of *en face* prepared vessels (for detailed description, see Supplementary Methods). The LSV endothelium was imaged *en face*, using confocal microscopy (Leica, Germany) and image analysis was all performed in 3D, either using Imaris (Supplementary Figure 3) or ImageJ (Supplementary Figure 4).

### Ex vivo monocyte adhesion

Following a 3-h incubation with 50 µmol/L NF-κB inhibitor, BAY11-7085, and exposure to acute arterial WSS for 6 h, LSV segments were dissected longitudinally, pinned with the luminal surface facing upwards and co-cultured, in static conditions, with 1 × 10^6^ Calcein AM-labelled (10 µmol/L) THP-1 cells for 15 min. Immediately after co-culture and washing, in situ adhered monocytes and LSV segments were imaged using fluorescence microscopy (Zeiss, Germany).

### Intimal RNA extraction

For extraction of RNA from LSV ECs, a technique was used which involves the flushing of the lumen of the vessel with a severe lysis buffer, in this case, Qiazol (Qiagen), to disrupt cells in the intimal layer, which were predominantly ECs^34^. Briefly, segments were cut transversely into 2–3 cm lengths and washed in ice-cold DPBS. Using a 1 mL syringe and 18-gauge unbevelled needle with the tip inserted a into the vessel lumen, the vessel was flushed with ice-cold DPBS. Finally, the lumen was quickly flushed (3–6 s) with 350 μL of ice-cold Qiazol and the eluate was collected in a 1.5 mL Eppendorf tube. This eluate was subjected to the RNA isolation protocol described later.

### EC culture and shear stress

HUVECs were exposed to laminar, unidirectional shear stress (at 0.5 or 12 dyn/cm^2^ to simulate venous and arterial rates of shear stress, respectively) for varying times, using parallel plate flow chambers designed in-house (Supplementary Figure 2), as described previously^35^, or maintained in static conditions. Following HUVEC dynamic culture for variable time-points, cells were subjected to analysis for immunocytochemistry, qPCR or Western Blotting.

### In vitro real-time monocyte adhesion

HUVECs were exposed to acute arterial WSS using the microfluidic capillary Bioflux 200 system, which allowed for dynamic co-culture with THP-1 cells and visualisation in real-time (Supplementary video 4). Briefly, following a 3 h incubation with 20 µmol/L NF-κB inhibitor, BAY11-7085, CmDiI-labelled HUVECs were exposed to acute arterial WSS at 12 dyn/cm^2^ for 4 h, after which point, Calcein-labelled THP-1 cells were introduced into the system and co-cultured, under shear stress (at 1 dyn/cm^2^), for a further 10 min, and imaged in real time (for detailed description, see Supplementary methods). Total number of adhered monocytes were then enumerated.

### Immunocytochemistry

Briefly, HUVECs cultured on glass slides and subsequently exposed to shear stress, or maintained in static conditions, were fixed in 3% Paraformaldehyde (PFA). Following fixation, HUVECs were stained with primary antibodies against either NF-κB, CCL2 or VE-Cadherin, followed by appropriate secondary antibodies. Slides were then imaged using the Zeiss AxioObserver Z1 fluorescent microscope and automated quantification was performed with CellProfiler analysis software (for detailed description, see supplementary methods).

### NF-κB activity assay

Total HUVEC lysates were used to detect NF-κB binding activity using a TransAM p65 DNA-binding ELISA kit (Active Motif, USA). The manufacturer’s instructions were followed and the colourimetric reaction endpoint was read at 450 nm.

### Statistical analyses

For experiments where only two groups were analysed, data were subjected to a paired, two- tailed t-test. For experiments where more than two groups were analysed, a one- or two-way ANOVA was used depending on the number of independent variables, followed by post-hoc pairwise comparisons with Bonferroni correction for multiple comparisons. If datasets were large enough (for example for immocytochemical analyses, where 20 images per sample were analysed), normal distribution was assessed with the D’Agostino-Pearson test; all data assessed passed normality tests, as such, parametric analyses were appropriate. The cut-off value for statistical significance was 0.05. Data are presented as mean ± SEM. All statistical analysis was performed with GraphPad Prism 7.0.

## Supplementary information

Supplementary Information 1.

Supplementary Video S1.

Supplementary Video S2.

Supplementary Video S3.

Supplementary Video S4.

## Abbreviations

CABG: Coronary artery bypass grafting
IH: Intimal hyperplasia
IHD: Ischaemic heart disease
NF-κB: Nuclear factor-κB
VEC: Venous endothelial cells
LSV: Long saphenous vein
CCL2: Monocyte chemoattractant protein 1
HUVEC: Human umbilical vein endothelial cells
IL-6: Interleukin-6
IL-8: Interleukin-8
ICAM-1: Intercellular adhesion molecule 1
