# Supplementary material for: NF-κB inhibition prevents acute shear stress-induced inflammation in the saphenous vein graft endothelium
Source: Sci Rep. 2020 Sep 15;10:15133. doi: 10.1038/s41598-020-71781-6 (PMC7492228; doi:10.1038/s41598-020-71781-6)
Supplement: Supplementary file 1 — Supplementary Information 1. [file 41598_2020_71781_MOESM1_ESM.docx]

NF-κB inhibition prevents acute shear stress-induced inflammation in the saphenous vein graft endothelium

**Alexander O. Ward^1^, Gianni D. Angelini^1^, Massimo Caputo^1^, Paul C. Evans^2^, Jason L. Johnson^1^, M. Saadeh Suleiman^1^, Robert M. Tulloh^1^, Sarah J. George^1^* and Mustafa Zakkar^1^*^#^**

Running Title: *NF-kappa B drives vein graft EC inflammation*

*Joint Senior Authors

^1^ Bristol Medical School, University of Bristol, Research Floor Level 7, Queens’ Building, Bristol Royal Infirmary, Upper Maudlin Street, Bristol, BS2 8HW, UK

^2^ Department of Infection, Immunity and Cardiovascular Diseases, University of Sheffield, Sheffield, S10 2TN, UK

# To whom correspondence should be addressed: Prof Mustafa Zakkar, Bristol Medical School, Research Floor Level 7, Queens’ Building, Bristol Royal Infirmary, Upper Maudlin Street, Bristol, BS2 8HW, UK; Email: mz207@le.ac.uk.ac.uk; Tel: +44 (0)116 2583019

**Keywords:** Haemodynamics; Anti-inflammatory; Venous bypass grafts; CCL2; Monocytes

**Supplementary data:** Supplementary figures 1-10; Supplementary table 1; Supplementary videos 1-4 (Legends only); Supplementary methods

**
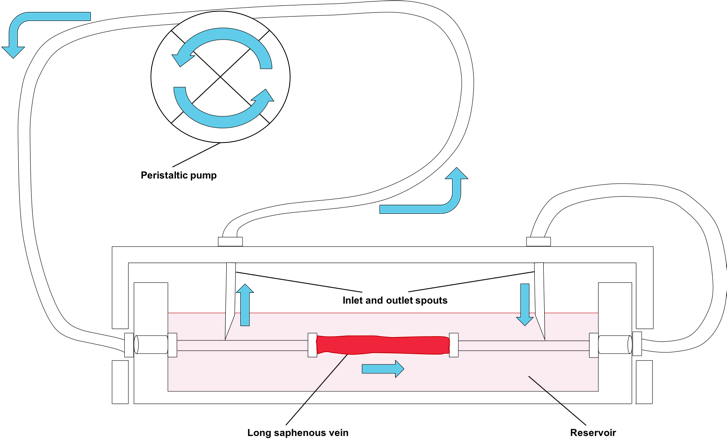
**

**A**


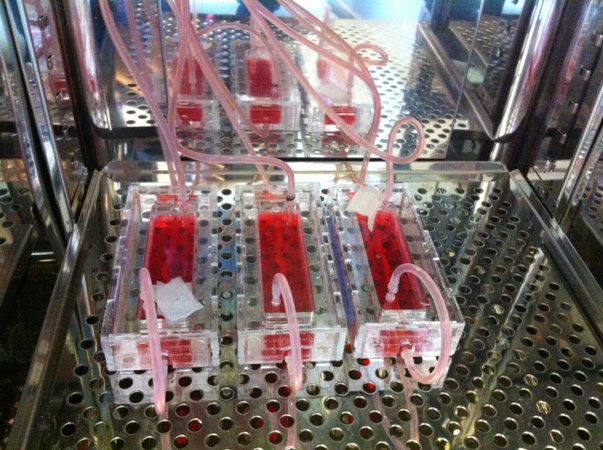


**B**

**Supplementary figure 1. *Ex vivo* perfusion system**

(A) Cross section of ex-vivo perfusion system. (B) Image of 3 ex vivo perfusion rigs for exposure of LSV to regulated levels of shear stress using a peristaltic pump.

**
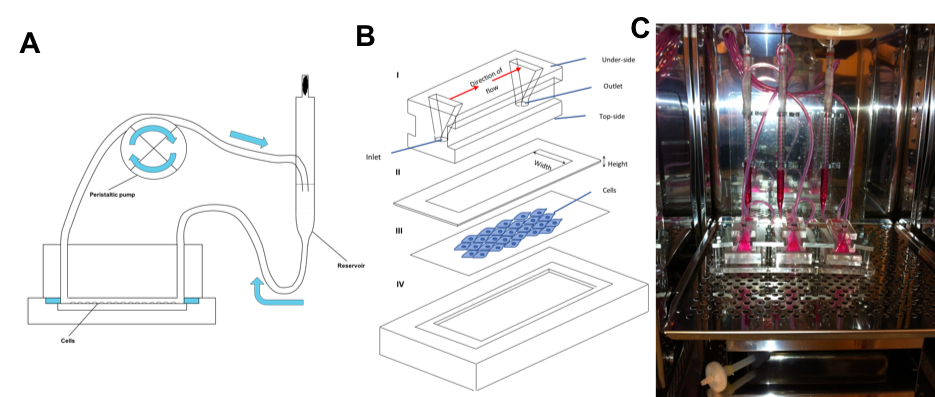
**

**Supplementary figure 2. *In vitro* parallel plate flow chamber (PPFC) apparatus**

(A) Cross-sectional view of PPFC with reservoir and tubing attached to peristaltic pump. (B) Schematic 3D diagram of PPFC: top section of flow rig containing ports and openings for both inlet and outlet (image drawn up-side down to visualise flow openings) (I), Silicon gasket to seal and provide barrier for flow area (II) and providing known width and height for calculation of shear stress, glass slide with cultured HUVECs (Blue) (III) and base plate for glass slide (IV). (C) Image of functioning PPFCs *in situ*, inside tissue culture incubator.


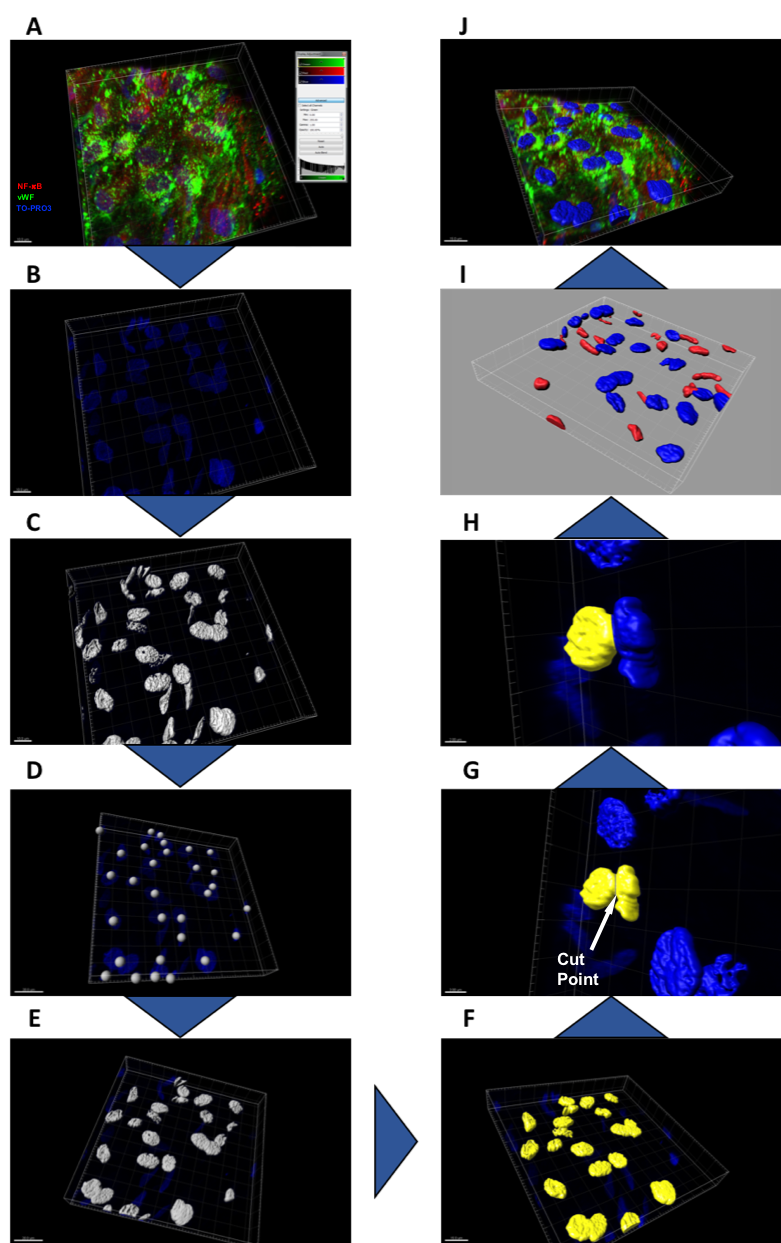


**Supplementary figure 3. 3D *en face* NF-κB immunofluorescence analysis process**

Imaris utilises 3D volumetric images (Z-stacks used here) to allow segmentation and quantification of images. The process represented here demonstrates the workflow undertaken for quantitative, volumetric analysis of solely the ECs of the LSV, whilst removing the VSMCs and other nuclei underneath the endothelium, from the analysis. (A) 3D volume reconstruction – En face prepared LSV, with all 3 channels represented in volume (Red: NF-κB; Green: vWF; Blue: TO-PRO3 (nuclear stain)). (B) 3D volume reconstruction – Nuclear stain only. (C) Surface rendering – Creation of surface rendered and smoothed nuclei (Grey) for segmentation. (D) Classify Seed points and Split touching objects – Semi-automated object detection allowing user defined inclusion or exclusion of objects based on ‘Quality’ or intensity of objects – each object should be individually represented by a grey sphere (set to 6μm for seed point (approx. size of nuclei)). (E) Classify surfaces – Removal of nuclei based on a morphological or intensity-based characteristic, in this case, oblate ellipticity (i.e. flat and elongated compared to a spheroid). (F) Surface selection – Create new surface rendered selection with only objects of interest. (G) Cut surfaces – Manual removal or clipping of objects – line in the yellow object (two adjoined nuclei), represents user defined ‘cut’ line (white arrow pointing to thin black line in the centre of the adjoining nuclei) for separation. (H) Cut surfaces – Separated objects – now considered as two independent nuclei. (I) Two distinct sets of nuclei segmented, blue representing EC nuclei, red representing other. (J)Final image to represent selected surface rendered objects on original image – Intensity and morphology statistics for all 3 channels to be exported from selected/segmented objects only.


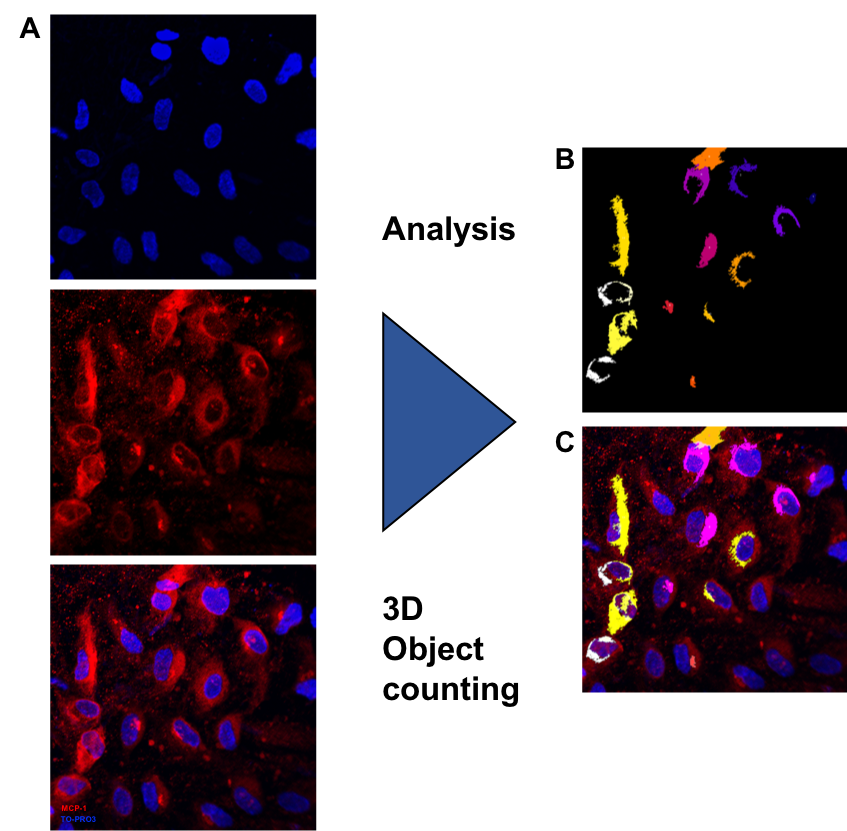


**Supplementary figure 4. FIJI-based *en face* CCL2 immunofluorescence analysis**

FIJI ‘3D Object counter’ uses Z-stacks as its input to segment objects that cross the user-defined intensity threshold. (A) Column of images represent maximum intensity projections (MIP) from Z-stacks of CCL2 (red) and nuclei (blue). (B) MIP of coloured 3D object map calculated by FIJI (colour coding is randomly assigned). (C) Overlay of en face immunofluorescence and 3D object map.


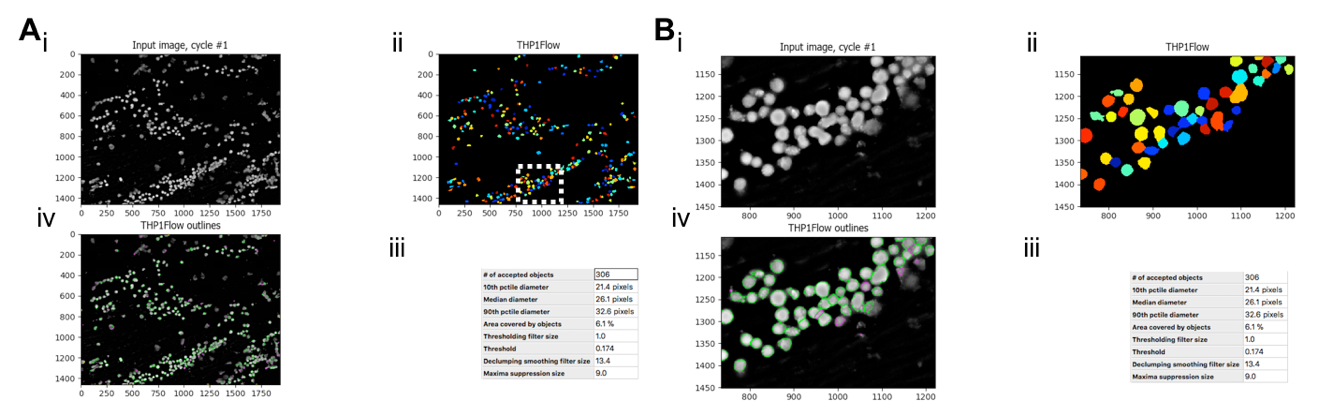


**Supplementary figure 5. *Ex vivo* monocyte adhesion enumeration**

(A) Identify primary objects – Identification and counts of labelled monocytes. Magnified region represented by dotted white box. (B) Magnification of A


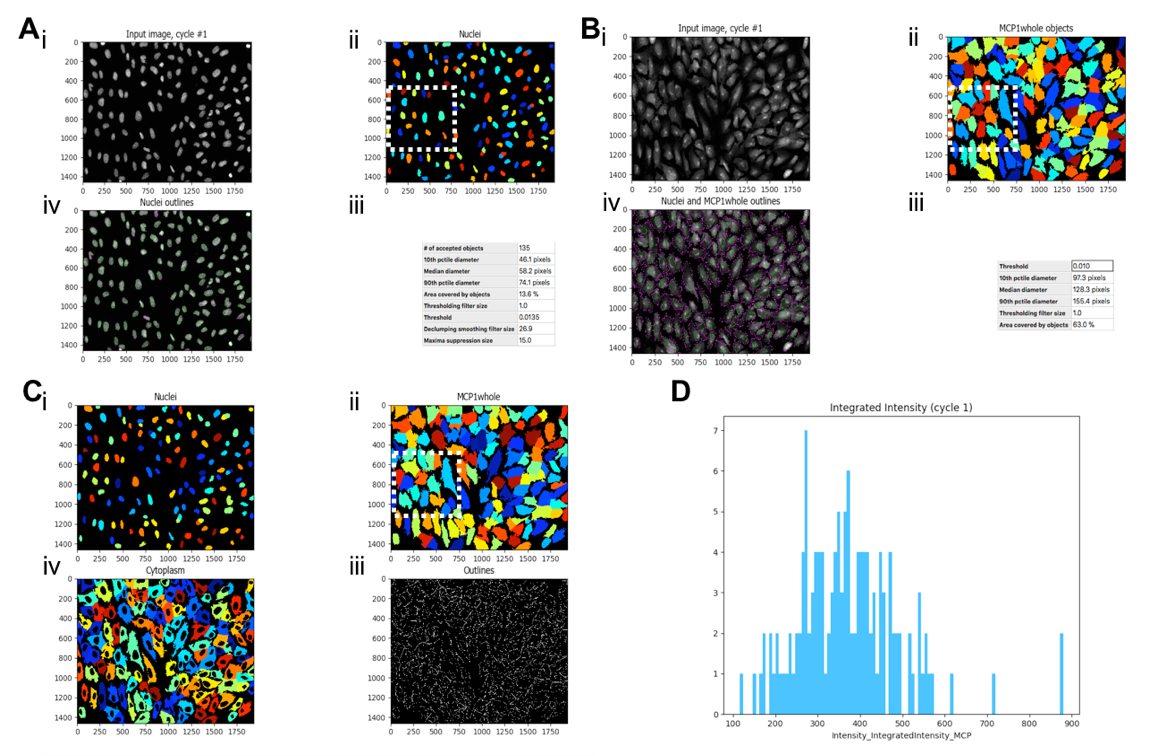

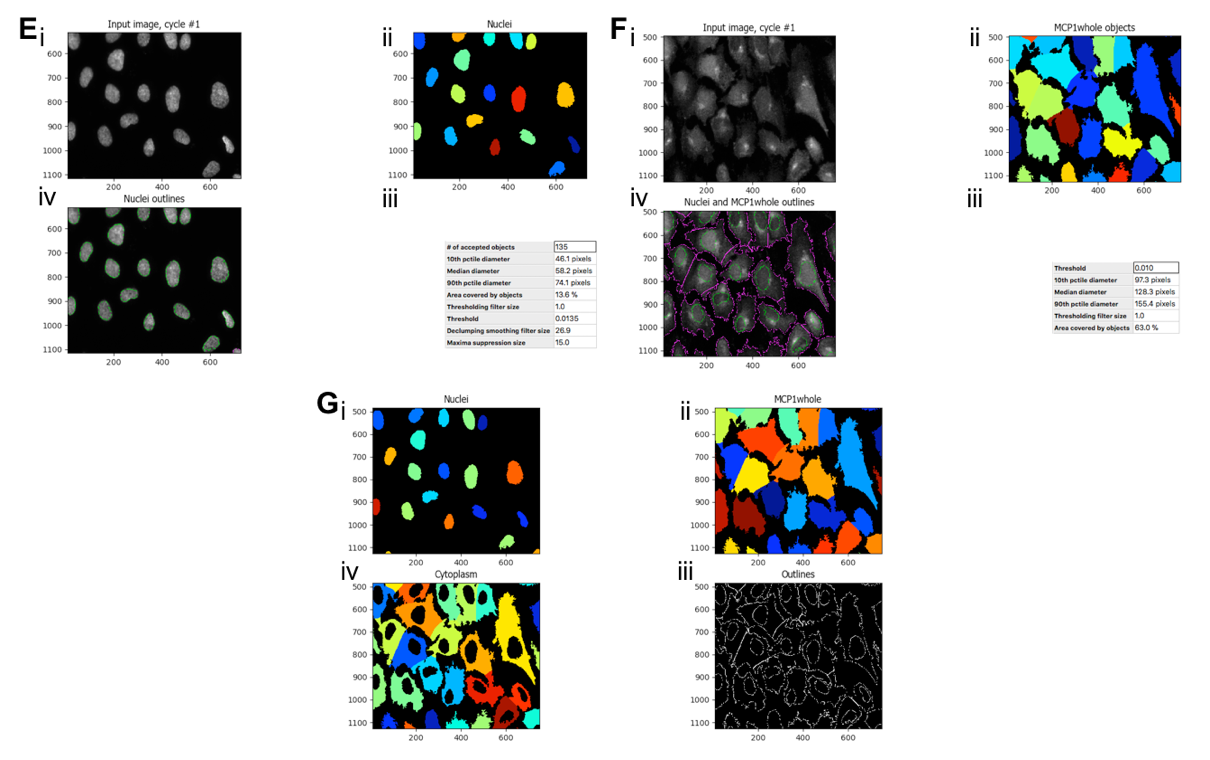


**Supplementary figure 6. *In vitro* CCL2 immunocytochemistry analysis**

CellProfiler was used for automated analysis of CCL2 fluorescence, outputs of the analysis are shown here to prove validity of method. Panels include grayscale original image (i), colour representation of image (i), quantitative outputs or outlines (iii) and original image with outlines (iv) clockwise from top left. (A) Identify Primary objects – Identification of seed regions from DAPI nuclear stain. (B) Identify Secondary objects – Grows from seed region to boundary of cells from using CCL2 immunostaining and step A as the seed. (C) Identify tertiary objects – Subtracts nuclei from whole cell to give cytosol segmentation. (D) Display Histogram – Outputs integrated intensity in the form of histogram for normality. White dotted box in A, B and C represents magnified area for E, F and G, respectively

**
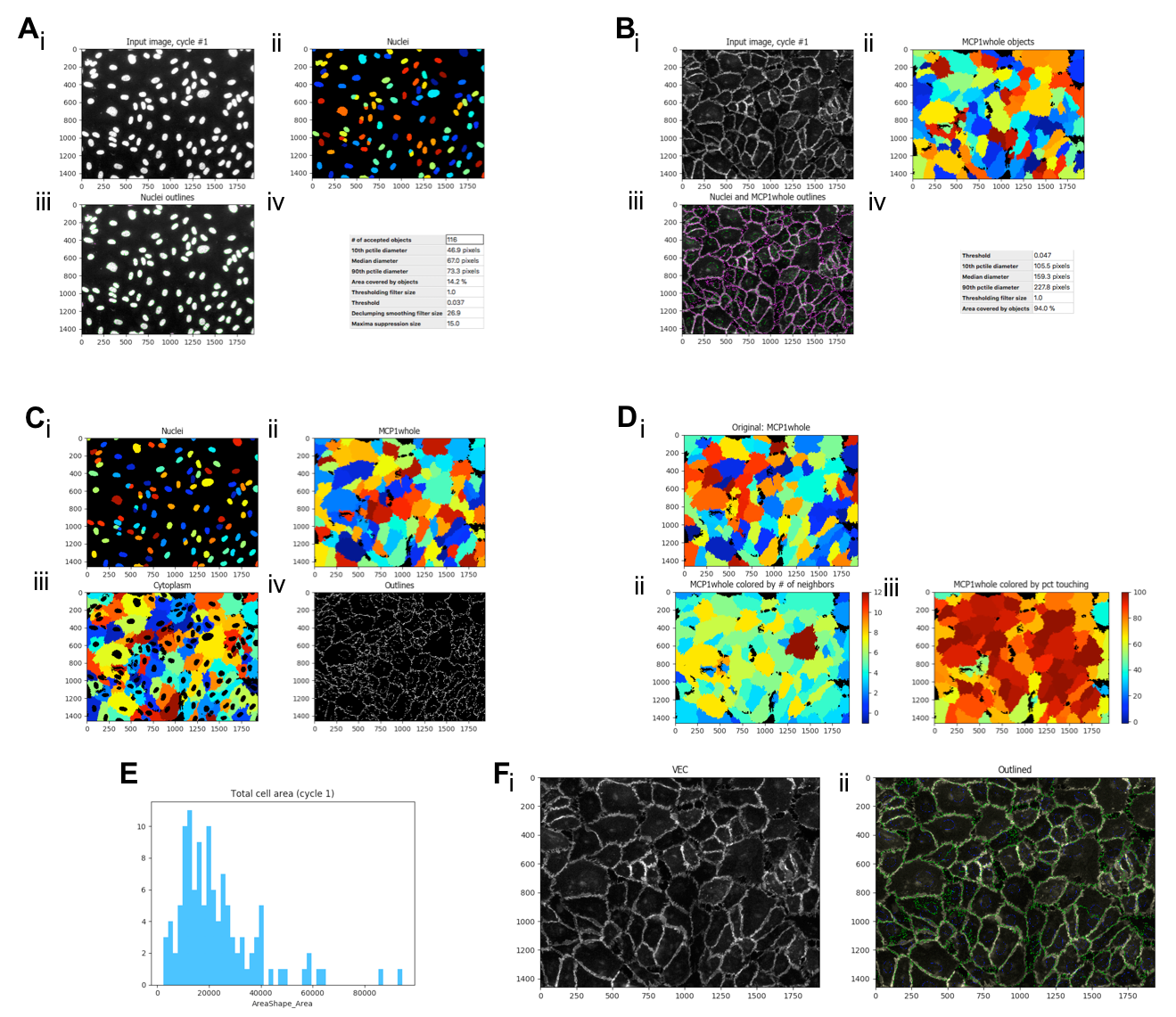
**

**Supplementary figure 7. *In vitro* VE-Cadherin cell-cell contact immunocytochemistry analysis**

Clockwise from top left, panels include grayscale original image (i), randomly assigned colour representation of image showing segmentation (ii), quantitative data outputs, visualisation of quantification or outlines (iii) and original image with outlines or image representing subtraction of nuclei from whole cell (iv). (A) Identify Primary objects – Identification of seed regions from DAPI nuclear stain. (B) Identify Secondary objects – Grows from seed region to boundary of cells from using VE-Cadherin immunofluorescence and step A as the seed. (C) Identify tertiary objects – Subtracts nuclei from whole cell to give cytosol segmentation. (D) Measure object neighbours – Measurement outputs including number of adjacent object neighbours (iii) and percent touching by object (iv). (E) Display Histogram – Outputs histogram of total cell area to show distribution. (F) Display outlines – Final representation of original image (i) and original image complete with nuclear outline (blue) and outer border outline (green) superimposed.


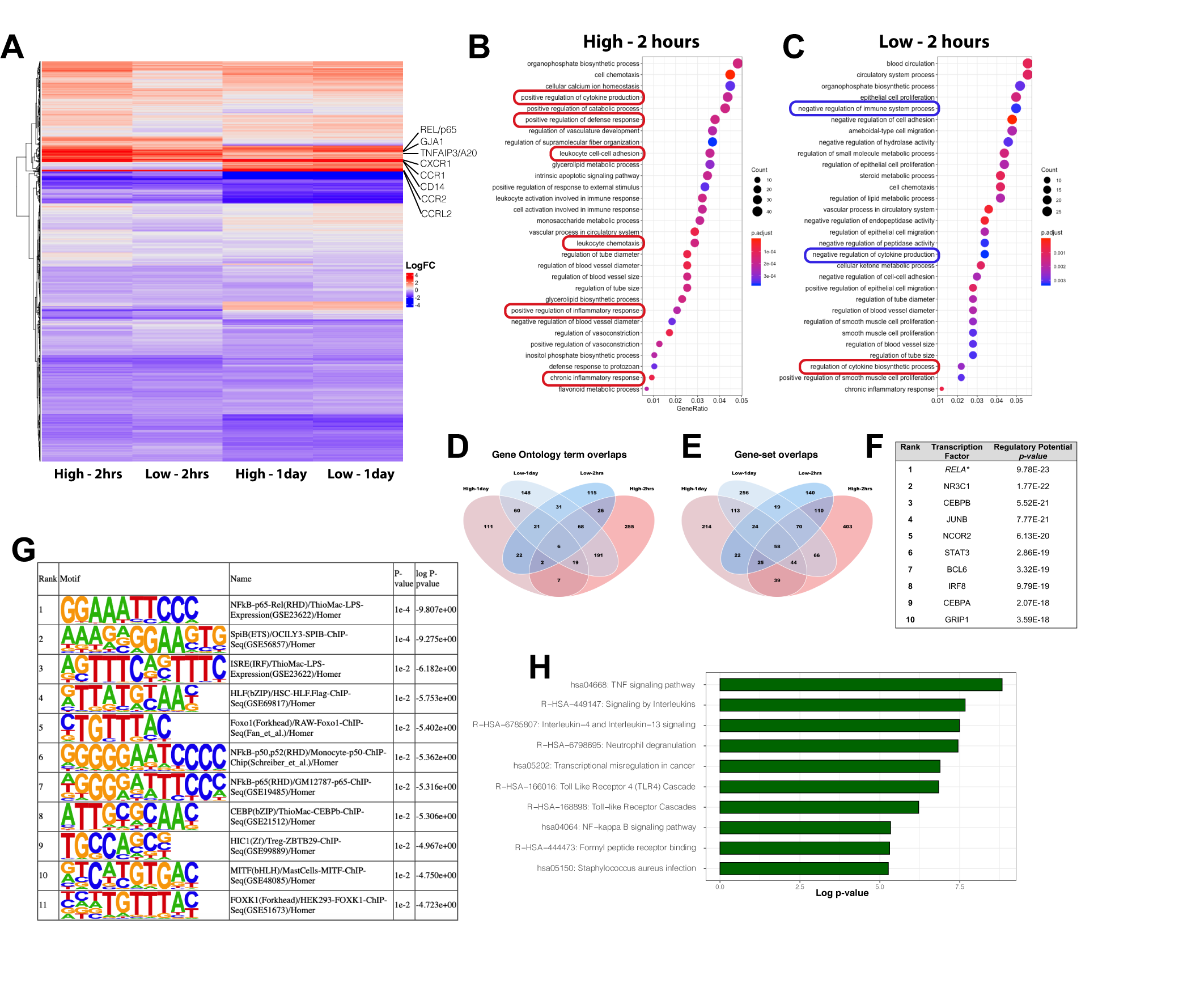


**Supplementary figure 8.**

Bioinformatic analysis of differentially expressed genes in rabbit vein grafts exposed to low or high shear stress for 2 hours and 1 day, using a publicly available dataset^4, 5^. (A) Gene expression data from a rabbit bilateral vein graft model showing differentially expressed genes (p<0.01 vs. Baseline) in any of the four graft conditions (High-2hrs, Low-2hrs, High-1day and Low-1day), with 7 of 16 NF-κB related genes amongst the most significantly different from baseline (indicated with black lines). (B) Differentially expressed (up-regulated) genes between Low-2hr and High-2hr grafts. (C) Top 20 most significant Biological process GO-terms enriched in Low-2hr and High-2hr grafts (vs. Baseline), obtained using the EnrichGO tool in Cluster Profiler. (D and E) Overlaps between enriched GO-terms and enriched gene-sets, respectively. (F) Transcriptional regulators of up-regulated genes, with a LogFC > 1.5 (114 genes), within High-2hr grafts were inferred using LISA and tabulated by Transcription Factor name and Regulatory potential p-value. (G) Known motif outputs from HOMER, shows significantly enriched motifs within the promoter of the top 114 genes, promoter regions were defined as between -1000 and +100bp from the transcription start site (TSS). (H) KEGG and REACTOME pathway analysis was performed on the top 114 genes and plotted by the log p-value of pathway enrichment.

**CCL2**

**Supplementary figure 9. *In vitro* validation of BAY11-7085 efficacy**

CCL2, VCAM-1, E-Selectin and IL-8 mRNA levels (A, B, C and D respectively) from HUVECs pre-treated with DMSO control (BAY11-7085 0μmol/L) or increasing concentrations of BAY11-7085 and then stimulated with 10ng/μL. mRNA expression data are normalised to β-tubulin and expressed as a fold-change relative to DMSO control. * indicates p<0.05, ** indicates p<0.01, *** indicates p<0.001, **** indicates p<0.0001, One-way ANOVA followed by post-hoc pairwise comparisons with Bonferroni correction for multiple comparisons, n=3.

**
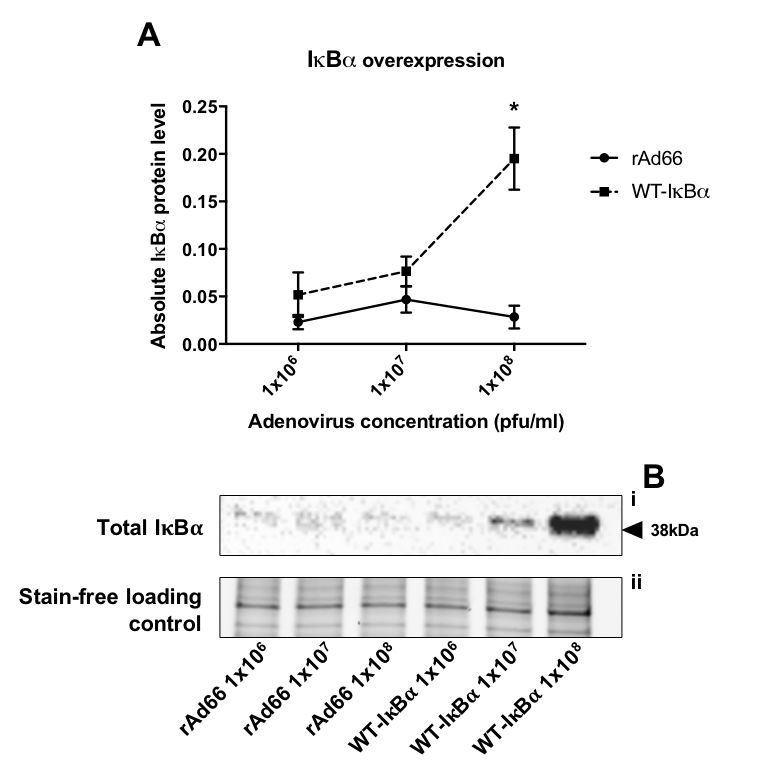
**

**Supplementary figure 10. Adenoviral-mediated overexpression of WT-IκBα**

Absolute IκBα protein levels were compared in HUVECs infected with WT-IκBα or rAd66 adenoviruses, by Western blot analysis. * indicates p<0.05, vs. rAd66 control, Two-way ANOVA followed by post-hoc pairwise comparisons with Bonferroni correction for multiple comparisons, n=3.

**
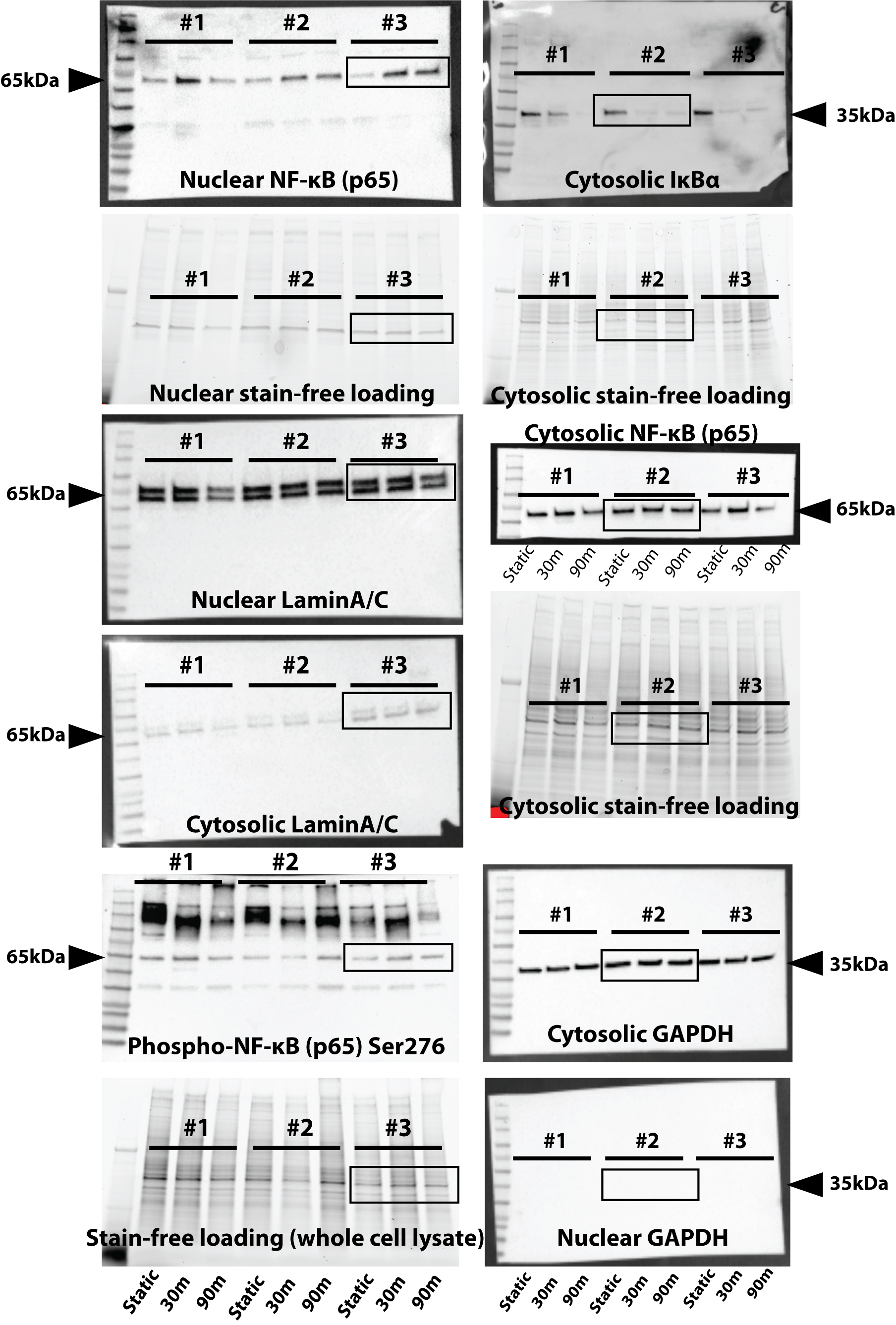
**

**Supplementary figure 11. Full length Western Blots and stain-free gels to accompany figure 3.**

WBs representative of 3 independent experiments are shown in their full length and unchanged versions. Black boxes correspond to the cropped samples shown in the main manuscript. Numbers 1, 2 and 3 correspond to 3 (of 3, 5 or 6) independent samples of HUVECs maintained in static conditions or exposed to shear stress for 30 or 90 minutes.


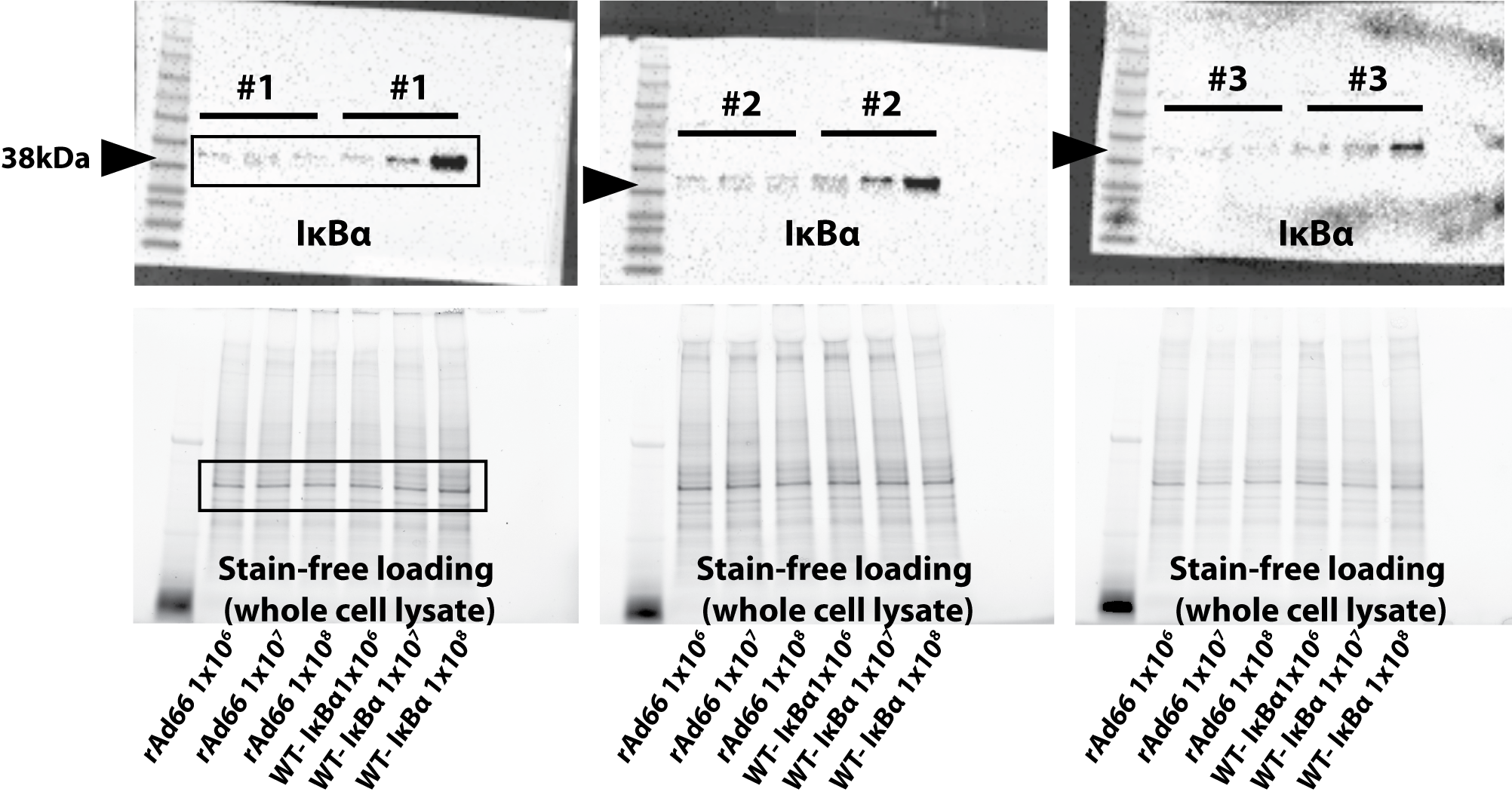


**Supplementary figure 12. Full length Western Blots and stain-free gels to accompany Supplementary figure 10.**

WBs representative of 3 independent experiments are shown in their full length and unchanged versions. Black boxes correspond to the cropped samples shown in the main manuscript. Numbers 1, 2 and 3 correspond to 3 independent samples of HUVECs transduced with varying concentrations (pfu/mL) WT- IκBα or empty viral vector control (rAd66), showing desired overexpression at the protein level.

**Supplementary Table**

| **GENE NAME** | **FORWARD SEQUENCE *(5’->3’)*** | **REVERSE SEQUENCE *(5’->3’)*** |  |
| --- | --- | --- | --- |
| Tubulin (TUBB1) | GCTGGACCGCATCTCTGTGTACT | TTACCTGCCCCAGACTGACCAAAT | |
| Monocyte chemoattractant  protein 1 (CCL2) | CTCAGCCAGATGCAATCAATGCCC | TTCTTTGGGACACTTGCTGCTGGT | |
| Interleukin 6 (IL-6) | AAATTCGGTACATCCTCGACGGCA | TTTTCACCAGGCAAGTCTCCTCAT | |
| Interleukin 8 (IL-8) | TGTGAAGGTGCAGTTTTGCCAAGG | AATTTCTGTGTTGGCGCAGTGTGG | |
| Intercellular adhesion molecule 1  (ICAM-1) | GCAGACAGTGACCATCTACAGCTT | GCCTCACACTTCACTGTCACCTC | |
| E-Selectin (SELE) | TCAGCTCTCACTTTGGTGCTTCTCA | TGCTGACAATAAGCACTGGCCTCA | |
| Vascular cell adhesion molecule 1  (VCAM-1) | GGCCCAGTTGAAGGATGCGGG | AGAGCACGAGAAGCTCAGGAGAA | |

**Supplementary Table 1. List of primer sequences**

**Supplementary Videos**

**Supplementary video 1. Z-stack of NF-κB *en face* immunofluorescence**

NF-κB protein immunofluorescence in the LSV endothelium was assessed using *en face* preparation and analysis of portions of the LSV. Supplementary video 1. shows a representative Z-stack from LSV immunofluorescence staining for NF-κB (red), vWF (green) and TO-PRO3/nuclei (blue) from a portion of LSV which was exposed to 30 minutes AHSS. The video pans from the top to bottom, showing first the ECs, marked by vWF co-staining (green), and then underlying VSMCs.

**Supplementary video 2. Z-stack of CCL2 *en face* immunofluorescence**

CCL2 protein immunofluorescence in the LSV endothelium, was assessed using *en face* preparation and immunofluorescent analysis of portions of the LSV. Confocal microscopy was used to acquire Z-stacked image sets at 5 equally-distributed points across the endothelial surface. Supplementary video 3. shows a representative Z-stack from LSV immunofluorescence staining for CCL2 (red), vWF (green) and TO-PRO3/nuclei (blue) from a portion of LSV which was exposed to 6 hours AHSS. The video pans from the bottom to top, showing first the underlying VSMCs and then ECs, marked by vWF co-staining (green).

**Supplementary video 3. Z-stack of CCL2 *en face* immunofluorescence with CCL2 segmentation overlaid**

As above, CCL2 protein immunofluorescence in the LSV endothelium was assessed using *en face* preparation and immunofluorescent analysis of portions of the LSV. Supplementary video 3. shows a representative Z-stack from LSV immunofluorescence staining for CCL2 (red), vWF (green) and TO-PRO3/nuclei (blue) from a portion of LSV which was exposed to 6 hours AHSS. Overlaid on the immunofluorescent images are the object maps from the segmentation of CCL2 protein, obtained using FIJI. The segmentation of objects for the same image is also represented in 2D in section 2.9.4. The video pans from the top to bottom, showing first the ECs, marked by vWF co-staining (green), and then underlying VSMCs. Co-localised with the CCL2 staining are the segmented objects in multiple colours.

**Supplementary video 4. Real-time monocyte adhesion to vECs *in vitro***

THP-1 monocytic-like cell adhesion to the vEC monolayer, *in vitro*, was performed in real-time using the Fluxion Bioflux 200. Briefly, HUVECs were pre-treated using the NF-κB inhibitor, BAY11-7085 (upper red channel in video), or DMSO (lower channel in video). Following which, they were exposed to AHSS for 6 hours and then dynamically co-cultured with THP-1 monocytes under shear stress for 10 minutes using the Bioflux microfluidic channel system, during this time sequential images were acquired (33milliseconds apart) to assess adhesion in real-time. Supplementary video 4. shows 10 minutes (sped up to one minute in length) of dynamic co-culture of THP-1 monocytes with ECs and subsequent adhesion of Calcein-labelled THP-1 monocytes (green) to HUVECs (red).

**Supplementary Methods**

***Ex vivo* perfusion of veins**

Surplus segments of surgically prepared human long saphenous vein (LSV), resected during coronary artery bypass graft surgery from anonymised consenting patients, were placed immediately in M199 GlutaMax medium supplemented with 10% (v/v) Foetal Calf Serum (FCS), 100µg/ml penicillin and 100U/ml streptomycin. The study was approved by the NRES Committee East of England – Norfolk ethics number (REC14/EE/1097), informed consent was obtained from all study participants prior to surgery and use of human tissue conformed to the principles outlined in the Declaration of Helsinki. Appropriate length sections were cut transversely for static/baseline control measurements and maintained in M199 culture medium; 6cm sections were cut for use in *ex vivo* perfusion. Vein sections were cannulated with Male Luer Fittings 1/16” (World Precision Instruments, USA) and secured with fine surgical tie. Secured veins were then attached to the perfusion circuit consisting of an in-house designed bioreactor, silicon tubing and a multi-channel peristaltic pump (Supplementary figure 1). During perfusion of vessels for between 30 minutes to 6 hours, the perfusion system was maintained at 37°C and 5% CO_2_. Using the diameter of the cannulae, shear stress through a non-deformable cylinder assuming laminar flow and the viscosity of water at 37°C, was calculated by the equation: τ=4µǪ/πr^3^. Where τ represents Shear Stress; µ, viscosity; Ǫ, flow rate; π, pi; r, radius. Hence, a flow rate of 100mL/min was required to achieve a wall shear stress of 12±0.2dyn/cm^2^. Veins were perfused with prewarmed M199 culture medium under mean arterial flow rate (100mL/min).

***En face* immunostaining**

Venous luminal LSV ECs were assessed for the localisation of NF-κB and quantity of CCL2 after exposure to AHSS by immunostaining of *en face* prepared portions of LSV. Veins were cut transversely into approximately 1-1.5cm lengths, washed once in DPBS and dissected longitudinally using fine forceps and fine spring scissors. Sections were pinned to dental wax using minuten pins with the luminal surface facing upwards, dental wax was arched slightly to tauten the luminal surface. It was then fixed using 4% (w/v) paraformaldehyde for 48 hours. Following fixation, tissue was maintained in Phosphate Buffered Saline (PBS) for microdissection with a stereomicroscope. Briefly, the intimal layer of the vein was peeled gently away from the adventitial and medial layers using fine forceps and, if necessary, further microdissection with spring scissors was performed to ensure thin, flat intimal sections for immunofluorescent analysis. Immunostaining of the free-floating intimal layer was performed in 24-well plates. Briefly, tissue was permeabilised for 16 hours at 4°C in 0.2% (v/v) Triton-X in PBS and then blocked with 20% (v/v) goat serum in PBS for one hour at room temperature (RT). For incubation with primary antibodies 1% Bovine serum albumin (BSA) in PBS was used, as follows: NF-κB/p65, rabbit anti-NF-κB/p65 (0.33μg/mL; Santa Cruz Biotechnology, sc-372) for 48 hours at 4°C; CCL2, rabbit anti-CCL2 (3.33μg/mL; Abcam, ab9669) for 48 hours at 4°C. vWF; Fluorescein Isothiocyanate (FITC)-conjugated sheep anti-vWF primary antibody (200μg/mL; Abcam, ab8822) for 24 hours at 4°C (refer to Major resources table for details). Anti-NF-κB/p65 and CCL2 primary antibodies were detected using goat anti-rabbit AlexaFluor 568 conjugated secondary antibody (1:200; Life Technologies) for 24 hours at 4°C. Nuclei were detected with far-red DNA binding probe, TO-PRO3 (ThermoFisher Scientific) for one hour at RT. Immunoglobulin matched controls at the same concentration as the primary antibodies were used as staining-specific negative controls. Samples were pinned at the four corners of the tissue with the luminal surface facing upwards, submerged in PBS and imaged with a confocal laser scanning microscope (Leica, Germany) using a water-dipping objective. Samples were imaged uniformly across the endothelial surface and Z-stacks were acquired at four points towards the outer regions of the four sides of the tissue (i.e. at the four points of a cross) and one taken centrally (Supplementary videos 1, 2 and 3). Z-stacks for NF-κB immunostaining were analysed by calculation of the mean fluorescence intensity inside the 3-D volume of EC nuclei, following removal of vascular smooth muscle cell (VSMC) nuclei based on their ellipticity in a semi-automated manner, using Imaris 3-D reconstruction software (Supplementary figure 3). Z-Stacks for CCL2 immunostaining were analysed using the ‘3D objects counter’ module in ImageJ, with a high fluorescence threshold (50 Arbitrary fluorescence intensity units (AFI)) and small lower object size (1000 voxels) (Supplementary figure 4).

***Ex vivo* monocyte adhesion**

Prior to exposure to AHSS, or maintenance under static conditions in M199 medium, segments of fresh LSV were cannulated, gently flushed with and submerged in 50µmol/L NF-κB inhibitor, BAY11-7085 (Santa Cruz Biotechnology, sc-202490), dissolved in DMSO (25mg/mL) or DMSO (0.5%v/v) vehicle control in M199 medium for 3 hours. Following a 6-hour exposure to flow using the perfusion system (Supplementary figure 1), veins were cut transversely into approximately 1cm portions. Vessels were then dissected longitudinally using fine forceps and fine spring scissors and pinned to a sterile glass petri dishes coated with sylgard silicone elastomer. Pinned samples were incubated with 1×10^6^ Calcein AM-labelled (10µM) THP-1 cells for 15 minutes maintained at 37°C in Roswell Park Memorial Institute-1640 (RPMI) Medium supplemented with 10% (v/v) FCS, 100µg/ml penicillin, 100U/ml streptomycin and 2μmol/L L-Glutamine, and then washed twice with DPBS. Veins were placed directly into Immersol W2010 aqueous immersion medium (Zeiss) on a glass slide with the luminal side facing downwards, a coverslip placed on top and imaged immediately, using the Zeiss AxioObserver Z1 fluorescent microscope. Samples were imaged at five equally-spaced points across the endothelial surface. Z-stacks were taken to account for the uneven and contoured surface of the LSV endothelium. Finally, the total number of adhered monocytes were counted per field of view using a Global, Otsu, Two-class threshold with Cell Profiler software (Supplementary figure 5).

**EC culture and shear stress**

Pooled Human Umbilical Vein Endothelial Cells (HUVECs) (from up to 4 donors) were purchased from Promocell (Germany) and cultured (≤Passage 4) to full confluency on glass microscope slides coated with 1% (v/v) gelatin (Sigma Aldrich, USA) with Ready-to-use Endothelial Cell Growth Medium (ECGM: Promocell). HUVECs were cultured overnight in Endothelial Cell Basal Medium (ECBM: Promocell) supplemented with 2% (v/v) FCS, 100μg/mL penicillin, 100IU/mL streptomycin and 2μmol/L L-Glutamine. HUVECs were then exposed to laminar, unidirectional shear stress (at 0.5 or 12 dyn/cm^2^ to simulate venous and arterial rates of shear stress, respectively) for varying times, using parallel plate flow chambers designed in-house (Supplementary figure 2), as described previously ^32^, or maintained in static conditions. Briefly, the glass slides were placed into the parallel plate chambers, and sealed with a silicon sheet gasket. A reservoir containing 30mL M199 GlutaMax culture medium, supplemented with 2% (v/v) FCS, 100µg/ml penicillin and 100U/ml streptomycin attached to a closed circuit loop of silicon tubing (VWR, USA and Elkay, UK) was connected to the chambers. HUVECs were then cultured at 37°C and 5% CO_2_ and shear stress applied using a multi-channel peristaltic pump (Watson-Marlow, UK). Shear stress rates were calculated for a slit die assuming the viscosity of water at 37°C, using the following equation: τ=6µǪ/wh^2^. Where τ represents Shear Stress; µ, viscosity; Ǫ, flow rate; w, width and h, height.

**THP-1 cell culture**

THP-1 human monocytic cells were purchased commercially (ATCC TIB-202), maintained in RPMI-1640 Medium supplemented with 10% (v/v) FCS, 100µg/ml penicillin, 100U/ml streptomycin and 2μmol/L L-Glutamine and passaged every 3-4 days, or until a maximal density of 1×10^6^ cells/ml was achieved.

**Overexpression of IκBα**

Overexpression of IκBα in HUVECs was achieved using adenoviral-mediated delivery of porcine wild type IκBα (Ad-WT-IκBα; provided by Dr Mark Bond, University of Bristol) as described previously^1^. Homology between the amino acid sequences of human and porcine IκBα is greater than 90% and the porcine IκBα adenoviral construct has in fact been found to be more effective in human ECs than porcine^1,2^. Briefly, HUVECs cultured on gelatin-coated glass slides were cultured using ECGM for 24 hours or until 70% confluency, and then infected with 1x10^8^ plaque forming units per mL (pfu/mL) of either Ad-WT-IκBα, or rAd66 empty vector control adenovirus, for 18 hours in ECBM. Medium containing the Adenovirus was replaced with ECBM supplemented with 2% FCS and antibiotics and ECs were cultured for a further 48 hours to achieve maximal transgene overexpression before exposing to shear stress and subsequent analyses.

**Immunocytochemistry**

HUVECs cultured on glass slides were exposed to shear stress or maintained under static conditions, then washed in ice-cold PBS and fixed in 3% Paraformaldehyde for 10 minutes. Following fixation, cells were washed 3 × 5 minutes in PBS. Briefly, cells were permeabilised for 2 × 5 minutes, using 0.1% (v/v) Triton-X in PBS, and blocked with 20% (v/v) goat serum in PBS for 45 minutes. Cells were then incubated with primary antibodies in 1% BSA/PBS, as follows: CCL2, rabbit anti-CCL2 primary antibody (3.33μg/mL; Abcam, ab9669) for 24 hours at 4°C, and bound primary anti-CCL2 antibodies were detected using a goat anti-rabbit AlexaFluor 568 fluorophore-conjugated secondary antibody for 1 hour at RT (1:200; Life Technologies, USA). VE-Cadherin, mouse anti-VE-Cadherin primary antibody (4μg/mL; Santa Cruz Biotechnology, sc-9989) for 1 hour at room temperature and bound anti-VE-Cadherin antibodies detected using a goat anti-mouse AlexaFluor 488 fluorophore-conjugated secondary antibody (1:200; Life Technologies) for 1 hour at RT. Immunoglobulin matched controls at the same concentration as the primary antibodies were used as staining-specific negative controls. Nuclei were labelled with diamidino phenylindole (DAPI)-dilactate (Invitrogen) in PBS for 1 hour. Finally, coverslips were mounted with Fluromount G (Life Technologies). Samples were imaged with the Zeiss AxioObserver Z1 fluorescent microscope. Quantification of fluorescence intensity was measured as integrated intensity in whole cells divided by total number of cells using CellProfiler 2.0^3^. Briefly, nuclei were segmented with a Global, Otsu, Two-class threshold based on nuclear size using DAPI staining. Outer cell boundaries were detected using CCL2 staining for each cell beginning with the identified nuclei as a reference by the ‘Watershed-Image’ detection method and Adaptive thresholding in CellProfiler (Supplementary figure 6). Finally, VE-Cadherin-based EC cell-cell contact structure was calculated as a percentage of objects (i.e. VE-Cadherin defined cell boundaries) in contact with one another, using a CellProfiler pipeline adapted from the one described above (Supplementary figure 7).

**Reverse Transcription Quantitative Polymerase Chain Reaction (RT-qPCR)**

mRNA transcript levels were quantified by RT-qPCR with gene-specific primers for CCL2, Interleukin-6 (IL-6), Interleukin-8 (IL-8) and Intercellular cellular adhesion molecule 1 (ICAM-1) (see Supplementary table 1). Extraction and purification of total RNA was performed using miRNeasy Mini kit (Qiagen, Germany), as per manufacturer’s instructions. Reverse transcription of total RNA was performed using transcriptor first strand cDNA synthesis kit (Roche, Switzerland). All RT-qPCR reactions were performed in triplicate. Expression of the specific target genes relative to the house-keeping gene, β-Tubulin, were calculated using the delta-delta CT method.

**Immunoblotting**

Total HUVEC protein was extracted with 1% (w/v) SDS lysis buffer, or, alternatively, cell compartment lysates were prepared with the NE-PER Nuclear and Cytoplasmic extraction kit (ThermoFisher). All Polyacrylamide Gel Electrophoresis (PAGE) were performed using the Bio-Rad Mini format 1-D electrophoresis system and 4-15% Mini-PROTEAN TGX stain free gels (Bio-Rad, USA). Gels were photoactivated using the ChemiDoc XRS+ imaging system (Bio-Rad) for endpoint normalisation of Western blot data to total protein concentration per lane. Following transfer, 0.2µM Nitrocellulose membranes (Bio-Rad), were blocked in 5% (w/v) semi-skimmed milk powder and primary antibodies were incubated overnight at 4°C (See supplementary methods for details). Primary antibodies were detected by Horseradish Peroxidase-conjugated secondary antibodies for 1 hour at RT and Luminata Forte Western HRP substrate reagent (Millipore, USA). Membranes were imaged using the ChemiDoc XRS+ imaging system and detected protein bands were quantified by densitometry and normalised to the stain-free loading control.

Primary antibodies used were as follows: NF-κB/p65, rabbit anti-NF-κB/p65 (0.1μg/mL; Santa Cruz Biotechnology, sc-372); phospsho-NF-κB/p65(Serine(Ser)276), rabbit anti-phospho-NF-κB/p65(Ser276) (0.4μg/mL; Santa Cruz Biotechnology, sc-101749); Inhibitor of NF-κB, alpha subunit (IκBα), rabbit anti-IκBα (0.4μg/mL; Santa Cruz Biotechnology, sc-371); Lamin A/C, rabbit anti-Lamin A/C (0.33μg/mL; Santa Cruz Biotechnology, sc-6215) for 1 hour at RT; Glyceraldehye 3-Phosphate dehydrogenase (GAPDH), mouse anti-GAPDH (3.33μg/mL; Millipore, MAB374)

***In vitro* real-time monocyte adhesion**

Prior to seeding HUVECs into the microfluidic channel system of the Bioflux 200 48-well, low shear plates (Fluxion; catalogue number: 910-0004), channels were coated with 1μg/mL bovine fibronectin (Sigma; catalogue number: F1141) for 1 hour at 37°C and 5% CO_2_. Unbound fibronectin was then removed from the wells and flushed out of the channels with ECGM. Using a cell density of 4x10^7^ cells/mL in a total volume of 10μL, 4x10^6^ HUVECs were seeded very quickly (2-4 seconds of flow) into the channels to prevent cell aggregation and blocking of microfluidic capillaries, whilst constantly viewing under the ThermoFisher EVOS FL microscope (ThermoFisher, AMF4300). Seeding was performed into the outlet well and HUVECs were flushed through the channels with a shear stress of 5dyn/cm^2^ for less than 5 seconds (until HUVECs entered the viewing channels). HUVECs were cultured in the channels for 48 hours at 37°C and 5% CO_2_. Following these 48 hours, and prior to exposure to high shear stress, ECs were labelled with 10μM CellTracker CM-DiI (ThermoFisher) red fluorescent probe for three-hours, concurrently with pre-treatment of HUVECs with either 20µmol/L NF-κB inhibitor BAY11-7085, or 0.2% (v/v) DMSO vehicle control at 37°C and 5% CO_2_. ECs were then exposed to unidirectional, pulsatile shear stress (at 12 dyn/cm^2^ and 1 hertz(Hz)) for 4 hours at 37°C and 5% CO_2_ using the Bioflux 200 system. THP-1 cells were labelled by incubation of 3x10^6^ THP-1 cells/mL with 10μmol/L Calcein AM (ThermoFisher) for 30 minutes, in a 6-well plate. Cells were then centrifuged at 300g for 5 minutes, to remove unbound Calcein AM, and cells were re-suspended at 3x10^6^ cells/mL. Following exposure of HUVECs to 6 hours of shear stress, Calcein AM-labelled THP-1 cells were flowed over the HUVECs (at 1dyn/cm^2^) for 10 minutes and viewing channels were imaged in real-time using a Zeiss AxioObserver Z1 inverted fluorescent microscope. Adhered THP-1 cells were enumerated after 10 minutes and divided by the total number of HUVECs in the viewing channels to calculate the percentage of adhered monocytes (see Supplementary video 4 for monocyte adhesion in real-time).

**Microarray analysis**

Microarray gene expression analysis was performed using a previously published and processed dataset^4, 5^. Briefly, gene expression microarray data was obtained using a rabbit bilateral interpositional vein graft model with branch ligation^6^, which results in the ligated graft being exposed to low shear stress (0.8±0.1dyn/cm^2^) and the contralateral graft being exposed to a high shear stress (12.4±1.1dyn/cm^2^) environment. The two time-points chosen from this dataset were 2 hours and 1-day post-implantation, both of which were compared to baseline. Differentially expressed genes from log expression values were determined in both low and high shear stress exposed grafts, at both time-points, against baseline, using a two-sided t-test, with custom-code written in R 3.5.2. and a statistical significance cut-off of p<0.01. Gene set enrichment analysis for gene ontology term mining in differentially expressed gene sets was performed using both the EnrichGO feature of Cluster Profiler^7^. Biological process GO terms (with a p-value above 0.01) and gene sets, were also directly compared between the four groups. To further investigate the involvement of the NF-κB pathway in the *in vivo* response to acute high shear stress, the most highly up-regulated genes (Log Fold Change > 1.5) were analysed for their inferred transcriptional regulators, using LISA, promoter analysis (-1000 and +100 around the TSS) for known motif enrichment using HOMER, and finally KEGG and REACTOME pathway analysis.

**References**

1. Wrighton CJ, HoferWarbinek R, Moll T, Eytner R, Bach FH and deMartin R. Inhibition of endothelial cell activation by adenovirus-mediated expression of I kappa B alpha, an inhibitor of the transcription factor NF-kappa B. Journal of Experimental Medicine. 1996;183:1013-1022.
2. Rupec RA, Poujol D, Grosgeorge J, Carle GF, Livolsi A, Peyron JF, Schmid RM, Baeuerle PA and Messer G. Structural analysis, expression, and chromosomal localization of the mouse ikba gene. Immunogenetics. 1999;49:395-403.
3. Carpenter AE, Jones TR, Lamprecht MR, Clarke C, Kang IH, Friman O, Guertin DA, Chang JH, Lindquist RA, Moffat J, Golland P, Sabatini DM. CellProfiler: image analysis software for identifying and quantifying cell phenotypes. Genome Biology. 2007; 7(10):R100
4. Wang Y, Xu M, Wang Z, Tao M, Zhu J, Wang L, et al. How to cluster gene expression dynamics in response to environmental signals. Brief Bioinformatics. 2013; 13(2):162–174
5. Casarin, S, Bercelli, SA, Garbey, M. Linking gene dynamics to vascular hyperplasia –Toward a predictive model of vein graft adaptation. PLoS ONE 12(11): e0187606.
6. Jiang Z, Wu L, Miller BL, Goldman DR, Fernandez CM, Abouhamze ZS, et al. A novel vein graft model: adaptation to differential flow environments. Am. J. Physiol. Heart Circ. Physiol. 2004; 286:240–245
7. Yu G, Wang L, Han Y, He Q (2012). “clusterProfiler: an R package for comparing biological themes among gene clusters.” OMICS: A Journal of Integrative Biology, 16(5), 284-287.
